# AAV-NRIP gene therapy ameliorates motor neuron degeneration and muscle atrophy in ALS model mice

**DOI:** 10.1186/s13395-024-00349-z

**Published:** 2024-07-24

**Authors:** Hsin-Hsiung Chen, Hsin-Tung Yeo, Yun-Hsin Huang, Li-Kai Tsai, Hsing-Jung Lai, Yeou-Ping Tsao, Show-Li Chen

**Affiliations:** 1 Graduate Institute of Microbiology, College of Medicine, National Taiwan University, Taipei, 100 Taiwan; 2 Department of Neurology, National Taiwan University Hospital, Taipei, 100 Taiwan; 3 Department of Ophthalmology, Mackay Memorial Hospital, Taipei, 104 Taiwan

**Keywords:** AAV, NRIP, Gene therapy, Motor neuron, Neuromuscular junction, Muscle, SOD1 G93A, ALS

## Abstract

**Background:**

Amyotrophic lateral sclerosis (ALS) is characterized by progressive motor neuron (MN) degeneration, leading to neuromuscular junction (NMJ) dismantling and severe muscle atrophy. The nuclear receptor interaction protein (NRIP) functions as a multifunctional protein. It directly interacts with calmodulin or α-actinin 2, serving as a calcium sensor for muscle contraction and maintaining sarcomere integrity. Additionally, NRIP binds with the acetylcholine receptor (AChR) for NMJ stabilization. Loss of NRIP in muscles results in progressive motor neuron degeneration with abnormal NMJ architecture, resembling ALS phenotypes. Therefore, we hypothesize that NRIP could be a therapeutic factor for ALS.

**Methods:**

We used SOD1 G93A mice, expressing human SOD1 with the ALS-linked G93A mutation, as an ALS model. An adeno-associated virus vector encoding the human *NRIP* gene (AAV-NRIP) was generated and injected into the muscles of SOD1 G93A mice at 60 days of age, before disease onset. Pathological and behavioral changes were measured to evaluate the therapeutic effects of AAV-NRIP on the disease progression of SOD1 G93A mice.

**Results:**

SOD1 G93A mice exhibited lower NRIP expression than wild-type mice in both the spinal cord and skeletal muscle tissues. Forced NRIP expression through AAV-NRIP intramuscular injection was observed in skeletal muscles and retrogradely transduced into the spinal cord. AAV-NRIP gene therapy enhanced movement distance and rearing frequencies in SOD1 G93A mice. Moreover, AAV-NRIP increased myofiber size and slow myosin expression, ameliorated NMJ degeneration and axon terminal denervation at NMJ, and increased the number of α-motor neurons (α-MNs) and compound muscle action potential (CMAP) in SOD1 G93A mice.

**Conclusions:**

AAV-NRIP gene therapy ameliorates muscle atrophy, motor neuron degeneration, and axon terminal denervation at NMJ, leading to increased NMJ transmission and improved motor functions in SOD1 G93A mice. Collectively, AAV-NRIP could be a potential therapeutic drug for ALS.

**Supplementary Information:**

The online version contains supplementary material available at 10.1186/s13395-024-00349-z.

## Background

Motor neuron diseases (MNDs) comprise a group of progressive neurological disorders that lead to the destruction of motor neurons, including amyotrophic lateral sclerosis (ALS), progressive muscular atrophy (PMA), and spinal muscular atrophy (SMA) [1]. ALS, the most common type of MND, typically manifests in adulthood (around 50–60 years old) [2]. It is a fatal neurodegenerative disease characterized by the progressive loss of both upper and lower motor neurons, resulting in death within 3–5 years after diagnosis [2, 3]. Although few treatments for ALS are available, none have a profound effect on survival [4–8]. ALS is epidemiologically classified into sporadic (90%–95%) and familial (5%–10%) forms (fALS); approximately 12% of fALS cases are caused by mutations in the SOD1 gene, leading to a toxic gain of function in the mutant protein [9, 10]. The human SOD1 G93A mutant transgenic mouse has become the most widely used ALS model, exhibiting features similar to human ALS [11, 12].

Several gene therapies for SOD1 mutant ALS have been developed to silence SOD1 mRNA expression, delivered in the form of antisense oligonucleotides (ASOs) or adeno-associated virus (AAV) vectors carrying short hairpin RNA (shRNA) [13, 14]. While most gene therapy for ALS is administered into the spinal cord via intrathecal injection to ameliorate neuronal pathology [15], studies indicate that ALS pathology follows a ‘dying back’ pattern, where muscle pathology precedes motor neuron death [16]. Therefore, some gene therapies for ALS have been developed via intramuscular injection to address muscle pathology and improve disease progression [1, 17, 18]. For example, AAV-insulin-like growth factor 1 (AAV-IGF1) injection into muscles has been shown to enhance motor activities and prolong the survival of SOD1 G93A mice [19]. Additionally, targeting neuromuscular junction (NMJ) components, such as Dok-7 and MuSK, has demonstrated therapeutic potential in preserving motor function [20–23]. Recently, Qalsody (tofersen), an ASO for silencing SOD1 G93A mRNA, has been approved as the only gene therapy for ALS by the U.S. Food and Drug Administration (FDA) [13].

Nuclear receptor interaction protein (NRIP), also known as DCAF6 and IQWD1, is an 860-amino acid protein with seven WD40 repeats and one IQ motif [24]. NRIP interacts with calcium/calmodulin (Ca^2+^/CaM) protein to regulate muscle contraction and with alpha-actinin-2 (ACTN2) for cardiomyocyte contraction [25–27]. NRIP is also located at the muscle NMJ, where it interacts with the acetylcholine receptor-alpha subunit (AChRα) for AChR complex stabilization [28]. Global NRIP knockout (NRIP gKO) mice exhibit skeletal muscle defects, resulting in reduced muscle contraction and delayed muscle regeneration [25]. Additionally, muscle-specific NRIP knockout (NRIP cKO) mice display NMJ degeneration, leading to retrograde α-motor neuron (α-MN) degeneration [29]. In muscle disorders, anti‐NRIP autoantibody presence correlates with myasthenia gravis (MG) severity [28]. However, the pathological significance of NRIP deficiency in MNDs remains unclear.

NRIP stabilizes NMJ structure and enhances muscle functions, thereby retrogradely supporting α-MN survival [25, 28, 29]. As muscle atrophy, NMJ degeneration, and α-MN death are pathological features of ALS [30], we hypothesized that NRIP could ameliorate these defects and enhance motor performance in ALS model mice. In this study, we generated AAV-NRIP (AAV-DJ/8 serotype) for gene therapy in SOD1 G93A mice via intramuscular injection. Our results demonstrate that AAV-NRIP increases myofiber size, reduces NMJ degeneration, and retrogradely supports α-motor neuron (α-MN) survival in SOD1 G93A mice. Consequently, AAV-NRIP enhances motor functions in SOD1 G93A mice by improving myofiber size, reducing NMJ degeneration, and supporting α-MN survival, leading to increased NMJ transmission. In summary, AAV-NRIP shows potential as a gene therapy for ALS.

## Results

### Low NRIP protein expression in the spinal cord and skeletal muscles of ALS model mice (SOD1 G93A)

**Fig. 1:**
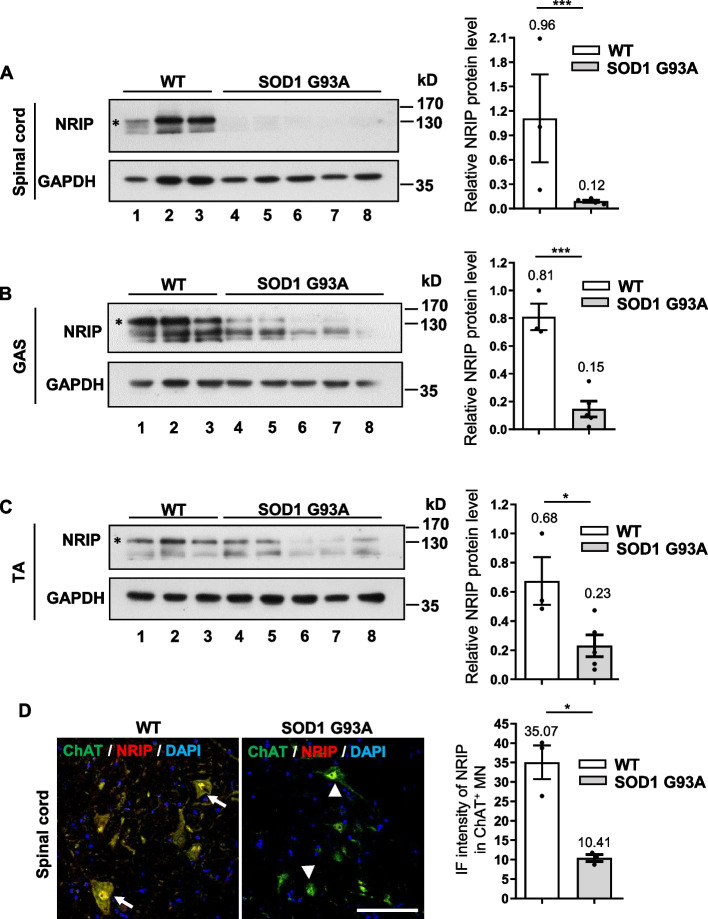
Low NRIP expression in the spinal cord and skeletal muscles of SOD1 G93A mice. **A** NRIP expression in the spinal cord at age of 56 days WT and SOD1 G93A mice. Mice were euthanized, and transcardial perfusion with PBS was performed to remove blood from tissues. Total proteins from L3-L5 spinal cord were subjected to Western blot (WB) analysis for NRIP expression, with GAPDH serving as the loading control. The asterisk indicates the representative band for NRIP expression. The right panel represents the quantification of NRIP expression conducted through densitometry analysis. **B** NRIP expression in the gastrocnemius (GAS) muscles. The asterisk indicates the representative band for NRIP expression. The right panel illustrates the quantification. **C** NRIP expression in the tibialis anterior (TA) muscles. The asterisk indicates the representative band for NRIP expression. The right panel shows the quantification. WT, *N* = 3 mice; SOD1 G93A, *N* = 5 mice. **D** NRIP expression in the lumbar segment of the spinal cord at the age of 56 days WT and SOD1 G93A mice. The 30 μm-thick sections were co-stained with anti-ChAT (green) and anti-NRIP (red) antibodies to detect NRIP expression in spinal cord motor neurons. DAPI was used as the nuclear counterstain. Arrows indicate high NRIP expression in ChAT-positive neurons, while arrowheads indicate low NRIP expression in ChAT-positive neurons. Scale bar: 100 μm. Right panel: Quantification of the immunofluorescence intensity of NRIP in ChAT-positive neurons from WT mice (*N* = 3) and SOD1 G93A mice (*N* = 3). Data are presented as mean ± SEM. Statistical analysis was conducted using the Student t-test. *P < 0.05 and ***P < 0.001

Due to NRIP being a multifunctional protein, it can directly interact with calmodulin (CaM) or α-actinin 2 in the presence of calcium [26, 27], playing a role in muscle contraction and maintenance of sarcomere integrity [25, 26]. Additionally, NRIP binds with AChR as a component of NMJ to retrogradely control motor neuron survival [28, 29]. Loss of NRIP in muscles causes motor neuron degeneration phenotypes similar to human and mouse ALS [31, 32]. To investigate whether NRIP can be a therapeutic target for ALS, this study utilized mice expressing human SOD1 (hSOD1) with the ALS-linked G93A mutation (SOD1 G93A mice) as an ALS model. Motor neuron and skeletal muscle tissues are the most vulnerable to ALS [2]. Firstly, NRIP protein expression (indicated by the asterisk) in the gastrocnemius (GAS) muscle, tibialis anterior (TA) muscle, and spinal cord from 56-day-old SOD1 G93A mice and age-matched wildtype (WT) mice were examined (Fig. 1A-C). The results showed that NRIP expression was low both in the spinal cord (Fig. 1A) and skeletal muscle tissues (Fig. 1B, C) of SOD1 G93A mice compared to WT mice. At age 8 weeks old, in the spinal cord, the relative NRIP protein level was significantly reduced in SOD1 G93A mice compared to WT mice (0.12 vs. 0.96, *P* < 0.001; Fig. 1A, right panel); and the gastrocnemius (GAS) and tibialis anterior (TA) also revealed decreased NRIP expression compared to WT mice (0.15 vs. 0.81, *P* < 0.001; 0.23 vs. 0.68, *P* < 0.05, respectively; Fig. 1B, C, right panel). To extensively analyze when NRIP starts to drop off in SOD1 G93A mice, we analyzed NRIP expression in 4-week-old and 6-week-old WT and SOD1 G93A mice. As shown in Figure S1, at 4 weeks of age, NRIP expression showed no difference between SOD1 G93A and WT mice in skeletal muscle and spinal cord tissues (Fig. S1A-C). However, at 6 weeks of age, the expression level of NRIP protein in SOD1 G93A mice began to decrease compared to WT mice in both skeletal muscle and spinal cord tissues (Fig. S1D-F). In the spinal cord at 6 weeks of age, the relative NRIP protein level was significantly reduced in SOD1 G93A mice compared to WT mice (0.77 vs. 1.01, *P* < 0.01; Fig. S1D, right panel). Similarly, the gastrocnemius (GAS) and tibialis anterior (TA) also showed decreased NRIP expression compared to WT mice (0.65 vs. 1.08, *P* < 0.05; 0.59 vs. 0.98, *P* < 0.05, respectively; Fig. S1E, F, right panel). Taken together, at 6 weeks of age, NRIP expression started to decline in SOD1 G93A mice in both muscles and spinal motor neurons. Some reported variation in pathogenesis begins in the SOD1 G93A mice [33]. Recently, it has been reported that the disease onset of SOD1 G93A mice is most commonly observed at postnatal day 90, when motor function starts declining [21, 33, 34], although some studies suggest that symptom onset occurs as early as day 60 [35]. The observed low NRIP expression in SOD1 G93A mice at 42 days, before motor function declined, suggests that downregulated NRIP expression in skeletal muscle and the spinal cord might contribute to ALS progression.

The roles of NRIP in motor neurons remain unclear. As shown in Fig. 1A, NRIP expression in spinal cords was lower in SOD1 G93A mice than in wild-type mice at 8 weeks of age. To further demonstrate NRIP expression within motor neurons, co-staining with anti-NRIP and anti-ChAT (a motor neuron marker) was performed. The immunofluorescence results showed that NRIP expression intensity in ChAT-positive motor neurons of SOD1 G93A mice was lower than in wild-type mice (Fig. 1D). This implies that NRIP could be expressed within motor neurons; combined with Fig. 1A, it suggests that NRIP expression within motor neurons of SOD1 G93A mice was declining. In the future, investigating the function of NRIP in motor neurons will be valuable.

### Forced NRIP expression in SOD1 G93A mice via AAV-mediated gene therapy

**Fig. 2:**
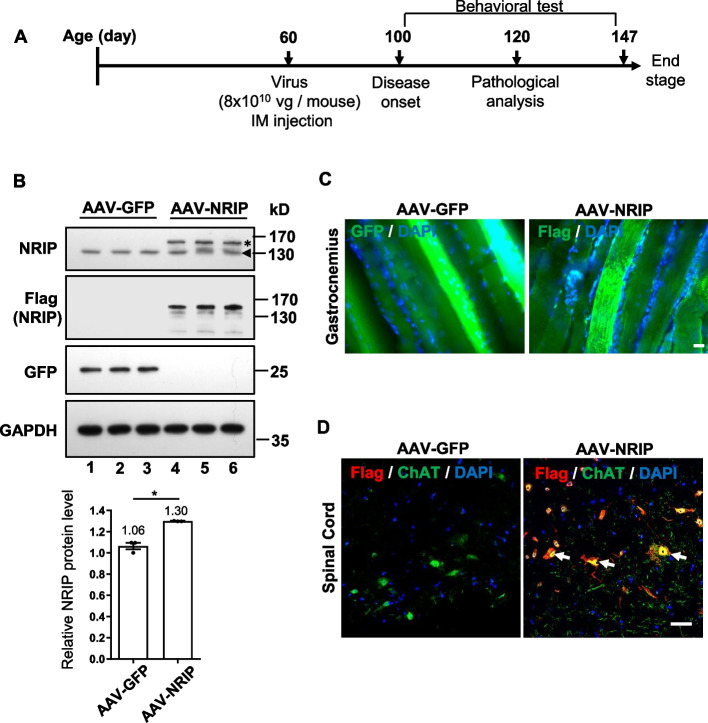
The gene therapy of SOD1 G93A via intramuscular injection of AAV-NRIP (**A**) Schematic representation of AAV-NRIP gene therapy. AAV-NRIP and AAV-GFP were delivered into the gastrocnemius (GAS) muscles of SOD1 G93A mice at postnatal day 60 through intramuscular injection. Behavioral tests were conducted from the age of 100 days (post-infection day 40) to 147 days (post-infection day 87). **B** Expression of NRIP in GAS muscles of AAV-NRIP treated SOD1 G93A mice. Protein extracts from the GAS muscles were collected at the end stage of the experiment and assessed for the expression of exogenous NRIP (Flag-NRIP) and GFP. The endogenous NRIP NRIP was detected by the anti-NRIP antibody. The asterisk indicated the Flag-NRIP expression; the arrowhead indicated the endogenous NRIP expression. GAPDH was the loading control. AAV-GFP group, *N* = 3 mice; AAV-NRIP group, *N* = 3 mice. **C** NRIP expression in muscles from AAV-GFP treated or AAV-NRIP treated SOD1 G93A mice. Immunofluorescence (IF) analysis for exogenous NRIP (right) expression with an anti-Flag antibody and GFP (left) expression with the anti-GFP antibody in GAS muscles. **D** NRIP retrograde expression in cholinergic neurons of the spinal cord from AAV-GFP treated or AAV-NRIP treated SOD1 G93A mice. IF stain for exogenous NRIP expression in the spinal cord. The Flag-NRIP expression was detected using an anti-Flag antibody (red), and the cholinergic neurons were stained using an anti-ChAT antibody (green). ChAT-positive neurons expressing Flag-NRIP are indicated by arrows. DAPI was the nuclear counterstain. Scale bars: 100 μm

To investigate the potential linkage between low NRIP expression in SOD1 G93A mice and progressive motor neuron degeneration, we aimed to assess whether forced NRIP expression in SOD1 G93A mice could have therapeutic effects through adeno-associated virus (AAV) gene therapy. AAV, a non-enveloped virus widely used for delivering DNA to target cells [36], was utilized in this study. The AAV DJ/8 serotype encoding Flag-NRIP was generated and named AAV-NRIP, with AAV-GFP serving as the control. The expression of Flag-NRIP and GFP was both under the control of the cytomegalovirus (CMV) promoter. The concentration of AAV-NRIP or AAV-GFP was 8 × 10^10^ vg/mouse. These were administered to the bilateral gastrocnemius (GAS) and tibialis anterior (TA) muscles of hindlimbs, as well as the bilateral triceps and biceps muscles of forelimbs in SOD1 G93A mice via intramuscular (im) injection. Considering the observed low NRIP expression at the age of 56 days in SOD1 G93A mice (Fig. 1), the AAV-gene therapy was initiated at postnatal day 60. Behavioral tests were performed at 100 days and continuously measured up to 147 days (see Fig. 2A for a timeline schematic). Pathological analyses, including NMJ number and innervation, as well as α-MN survival, were conducted at 120 days (post-injection day 60) (Fig. 2A).

It has been reported that AAVs exhibit retrograde trafficking activity from muscles to the entire spinal cord, including lumbar, thoracic, and cervical segments [37]. AAV retrograde transduction could occur either from the axon terminal endplates of injected muscles, which are located in the lumbar and cervical spinal cords, or via the bloodstream, potentially reaching the thoracic segments, which do not have the axonal endplates from the injected muscles [38]. Initially, we examined whether the intramuscular injection of AAV-GFP (AAV DJ/8 serotype) had a retrograde effect on the spinal cord. Wild-type (WT) mice were infected with AAV-GFP via intramuscular injection at the age of 60 days, and the cervical, thoracic, and lumbar segments of the spinal cord were collected at the age of 120 days. The sections of each segment were stained with anti-GFP (for GFP expression) and anti-NeuN (for neuron cells) antibodies to label the GFP-positive neuron cells. The results demonstrated that the control WT mice (no AAV-GFP treatment) showed no GFP expression in each segment of spinal cord (Fig. S2). In AAV-GFP treated WT mice, GFP proteins could be expressed in the lumbar, thoracic, and cervical segments of the spinal cord, with expression being strongest in the lumbar, gradually lower in the thoracic, and lowest in the cervical segment (Fig. S2). Consistent with previous reports, AAV DJ/8 could exhibit retrograde transfer from skeletal muscles to the entire spinal cord through axon terminal motor endplates or vascular spread.

On the other hand, SOD1G93A transgenic mice contain the human SOD1 with the G93A mutant form; hence, the transgene copy number would potentially affect the disease progression [23]. Before the treatment of AAV-NRIP and AAV-GFP, the transgene number of each group was examined by RT-PCR and shown to be comparable (Fig. S3). Subsequently, the exogenous NRIP (Flag-NRIP) in the gastrocnemius muscles of AAV-NRIP-treated SOD1 G93A mice at the age of 120 days was presented using an anti-Flag antibody for Western blot (Fig. 2B, lanes 4–6); AAV-GFP-treated mice showed GFP protein expression (Fig. 2B, lanes 1–3). NRIP is highly expressed in skeletal muscles, with endogenous NRIP located in the sarcomere and NMJ [25, 29]. Previously, we identified that NRIP can feed forward to activate its own promoter activity [39]. As shown in Fig. 2B, we used an anti-NRIP antibody to detect endogenous NRIP in AAV-GFP and AAV-NRIP-treated SOD1 G93A mice. The endogenous NRIP expression (arrowheads) in AAV-NRIP-treated SOD1 G93A mice was higher than in AAV-GFP-treated mice (Fig. 2B, lower panel), further supporting our previous report. The immunofluorescence analysis with an anti-Flag antibody also confirmed the presence of exogenous NRIP (Flag) in the gastrocnemius muscles (Fig. 2C). To determine the retrograde effect of AAV-NRIP on the spinal cord, the immunofluorescence stain in the lumbar segment of the spinal cord showed the exogenous NRIP coupled with choline acetyltransferase (ChAT)-positive motor neurons (Fig. 2D). This highlights that AAV-NRIP gene therapy could have an effect on both limb muscles and the lumbar spinal cord.

### AAV-NRIP gene therapy improves locomotor activity in SOD1 G93A mice

**Fig. 3:**
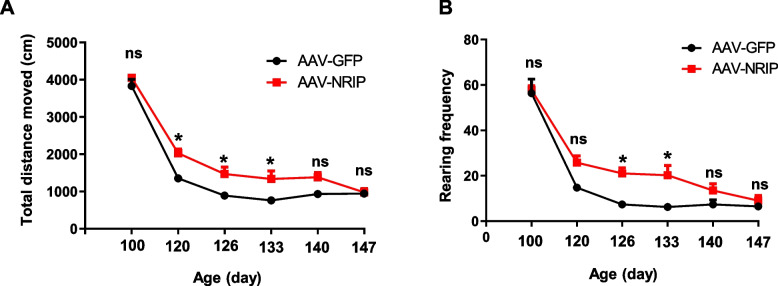
AAV-NRIP gene therapy improves locomotor activity in SOD1 G93A mice. **A** Total distance moved by SOD1 G93A mice with AAV-GFP or AAV-NRIP treatment. From the age of 100 days (post-infection day 40) to 147 days (post-infection day 87), the movement of the mice for 10 min was recorded using EthoVision Video Tracking Software (Noldus). The total distance moved (total distance mice traveled in the box) was measured during the test. Data are presented as mean ± SEM. *P = 0.019 at the age of 120 days; *P = 0.0452 at the age of 126 days; *P = 0.0494 at the age of 133 days; ns, no significance; one-way ANOVA with Tukey’s post hoc test. **B** Rearing frequency (frequency at which the mice stand on their hindlimbs in the box). The number of rearing was measured from the age of 100 days to 147 days. Data are presented as mean ± SEM. *P = 0.0496 at the age of 126 days; *P = 0.0382 at the age of 133 days; ns, no significance; one-way ANOVA with Tukey’s post hoc test

To investigate the therapeutic efficacy of AAV-NRIP on motor functions of SOD1 G93A, locomotor activity, grip force, and rotarod activity were measured at the indicated days. In locomotor activity, the total distance moved and rearing frequency were measured within an opaque acrylic box for 10 min. Rearing frequency is the number of times the mouse stood up on its hind legs within the open-top box within the period. AAV-NRIP-treated mice showed improvement in total distance movement from age 120 days (post-injection day 60) to 133 days (post-injection day 73) compared to AAV-GFP-treated mice (Fig. 3A). Rearing frequency was also enhanced in AAV-NRIP-treated SOD1 G93A mice from the age of 126 days (post-injection day 66) to 133 days (post-injection day 73) compared to AAV-GFP-treated mice (Fig. 3B). Similarly, males and females also presented a significant difference in total distance movement and rearing frequency between the AAV-NRIP treated group and the AAV-GFP treated group, respectively (Fig. S4A, B). However, the rotarod test showed that AAV-NRIP gene therapy had no effect on improving the retention time of SOD1 G93A mice on the rotating rod (Fig. S5A); and AAV-NRIP treatment had no effect on grip strength (Fig S5B). In summary, AAV-NRIP gene therapy could, at least, improve locomotor activity during disease progression in SOD1 G93A mice.

### Survival analysis of SOD1 G93A mice with AAV-NRIP gene therapy

Patients with ALS typically succumb to respiratory failure [2]. The disease onset in SOD1 G93A mice occurs around the age of 100 days, with the end stage of the disease defined as the day when mice are unable to right themselves within 15 s [40]. To assess whether AAV-NRIP gene therapy could extend the life of SOD1 G93A mice, the survival rates of AAV-GFP and AAV-NRIP-treated SOD1 G93A mice were monitored from the day of birth until the end stage. The Kaplan–Meier survival curve indicated that AAV-NRIP did not have a significant effect on the survival of SOD1 G93A mice (Fig. S5C). In summary, muscle-delivered AAV-NRIP successfully restored NRIP expression in skeletal muscle and the spinal cord, leading to an improvement in the locomotor activity of SOD1 G93A mice. However, there was no significant effect on life prolongation.

### Enhancement of myofiber size and slow myosin expression by AAV-NRIP gene therapy in SOD1 G93A mice

**Fig. 4:**
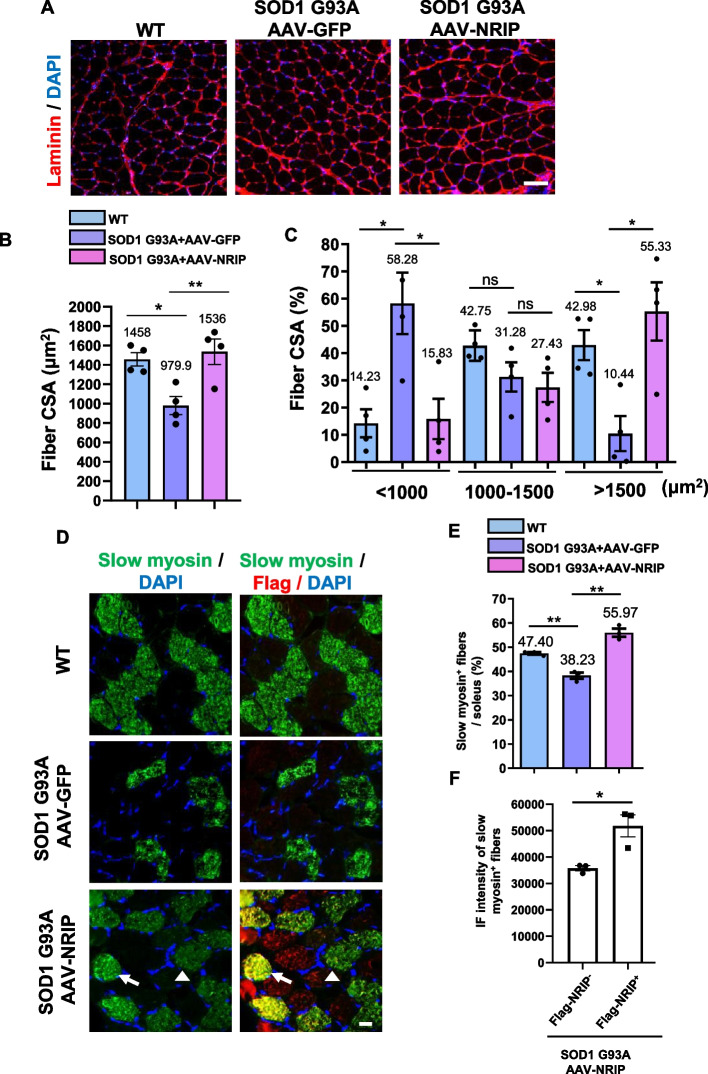
AAV-NRIP treated SOD1 G93A mice increase myofiber size and slow myosin expression. **A** Myofiber size in SOD1 G93A mice treated with AAV-NRIP. Soleus muscles were stained with anti-laminin (red) to define the muscle fiber borders. DAPI was used for nuclear staining. Scale bars: 50 μm. **B** The average myofiber cross-sectional area (CSA) was measured from WT and SOD1 G93A mice treated with AAV-GFP or AAV-NRIP. The CSA of all myofibers circumscribed by laminin was measured using ImageJ software (*N* = 4 for each group). Data are presented as mean ± SEM. *P = 0.0214 (WT vs. SOD1 G93A + AAV-GFP); **P = 0.0093 (SOD1 G93A + AAV-GFP vs. SOD1 G93A + AAV-NRIP); one-way ANOVA with Tukey’s post hoc test. **C** Distribution of different myofiber sizes in soleus muscles from WT and SOD1 G93A mice treated with AAV-GFP and AAV-NRIP. The CSA of myofibers was categorized into three groups based on size (< 1000 μm^2^, 1000–1500 μm^2^, or > 1500 μm^2^). The percentage of each categorized myofiber size relative to the total myofibers was calculated to assess the effect of AAV-NRIP on myofiber CSA (*N* = 4 for each group). Data are presented as mean ± SEM. *P = 0.0146 (< 1000 μm^2^, WT vs. SOD1 G93A + AAV-GFP); *P = 0.0168 (< 1000 μm^2^, SOD1 G93A + AAV-GFP vs. SOD1 G93A + AAV-NRIP); *P = 0.0390 (> 1500 μm^2^, WT vs. SOD1 G93A + AAV-GFP); *P = 0.0137 (> 1500 μm^2^, SOD1 G93A + AAV-GFP vs. SOD1 G93A + AAV-NRIP); one-way ANOVA with Tukey’s post hoc test. **D** AAV-NRIP increases slow myosin expression in the soleus muscle of SOD1 G93A mice. The soleus muscles were co-stained with anti-slow myosin and anti-Flag antibodies to visualize oxidative fibers (green) and Flag-NRIP expression (red). DAPI was used for nuclear staining. Scale bars: 50 μm. **E** Proportion of slow myosin-positive myofibers relative to the total myofibers (*N* = 3 for each group). Data are presented as mean ± SEM. **P = 0.0053 (WT vs. SOD1 G93A + AAV-GFP); **P = 0.0012 (SOD1 G93A + AAV-GFP vs. SOD1 G93A + AAV-NRIP); one-way ANOVA with Tukey’s post hoc test. **F** Fluorescence intensity of slow myosin in myotubes with or without Flag-NRIP expression in AAV-NRIP treated SOD1 G93A mice (*N* = 3 mice). Data are presented as mean ± SEM. Statistical analysis was conducted using Student t-test for panel F. *P < 0.05

SOD1 G93A mice exhibit reduced skeletal muscle masses and muscle cross-sectional areas, indicative of muscle atrophy [41–43]. NRIP is known to regulate myoblast differentiation during muscle regeneration [25]. To assess whether AAV-NRIP could augment the myofiber size in SOD1 G93A mice, soleus muscles from WT and AAV-treated SOD1 G93A mice at the age of 120 days were stained with an anti-laminin (red) antibody to delineate the muscle boundary (Fig. 4A). AAVs were injected into the bilateral hind limbs (gastrocnemius and tibialis anterior muscles) and forelimbs (triceps and biceps). However, adjacent muscles such as the soleus were also expressed by AAV-NRIP, as shown in Fig. 4D, where the tagged Flag (NRIP gene) was detected in the lower right panel. Therefore, we used the soleus muscles for the analysis of the cross-sectional area of myofiber size and slow myosin expression. The CSA of myofibers was measured based on the laminin-circumscribed muscle boundaries, as illustrated in Fig. 4A. The quantitative result of overall CSA showed that SOD1 G93A mice had a smaller fiber CSA than WT mice (979.9 μm^2^ vs. 1458 μm^2^,* P* < 0.05; Fig. 4B). AAV-NRIP could increase the fiber CSA in SOD1 G93A mice compared to the AAV-GFP (1536 μm^2^ vs. 979.9 μm^2^,* P* < 0.01; Fig. 4B). To investigate the effect of AAV-NRIP on CSA distribution, the fiber CSA was categorized into three groups of myofiber CSA (< 1000 μm^2^ or 1000–1500 μm^2^ or > 1500 μm^2^). Quantitative analysis revealed that AAV-GFP treated SOD1 G93A mice exhibited a higher proportion of myofibers with CSA < 1000 μm^2^ compared to WT mice (58.28% vs. 14.23%, *P* < 0.005; Fig. 4C), and a lower proportion of myofibers with CSA > 1500 μm^2^ than WT mice (10.44% vs. 42.98%, *P* < 0.05; Fig. 4C). In contrast, AAV-NRIP treatment increased the proportion of myofibers with CSA > 1500 μm^2^ in SOD1 G93A mice compared to AAV-GFP treated SOD1 G93A mice (55.33% vs. 10.44%,* P* < 0.05; Fig. 4C) and decreased the proportion of myofibers with CSA < 1000 μm^2^ in SOD1 G93A mice compared to AAV-GFP treated mice (15.83% vs. 58.28%, *P* < 0.05; Fig. 4C). Hence, AAV-NRIP treatment could increase myofiber size in SOD1 G93A mice.

The soleus muscle in SOD1 G93A mice has been reported to undergo a transition from slow-twitch to fast-twitch myofibers, contributing to motor neuron degeneration and muscle denervation [44, 45]. Given that NRIP can induce slow myosin expression via the Ca^2+^/CaM pathway [25], the expression of slow myosin in soleus muscles from AAV-NRIP infected SOD1 G93A mice was analyzed by immunofluorescence intensity of slow myosin staining (Fig. 4D). The results demonstrated that the reduction of slow myosin in SOD1 G93A mice was rescued by AAV-NRIP treatment (Fig. 4E). The AAV-NRIP treated SOD1 mice showed a higher percentage of slow myosin expression than WT mice (Fig. 4E), possibly due to the constitutive expression of Flag-NRIP driven by the CMV promoter. Furthermore, to examine the autonomous effect of NRIP on slow myosin expression within AAV-NRIP infected myofibers, co-staining results revealed that Flag-NRIP-positive myofibers (Fig. 4F, marked by arrow) exhibited higher intensity of slow myosin expression than Flag-NRIP-negative myofibers (Fig. 4F, marked by arrowhead). In summary, AAV-NRIP gene therapy can ameliorate the reduced myofiber size and increase slow myosin expression in SOD1 G93A mice.

### Amelioration of NMJ degeneration and axon terminal denervation by AAV-NRIP gene therapy in SOD1 G93A mice

**Fig. 5:**
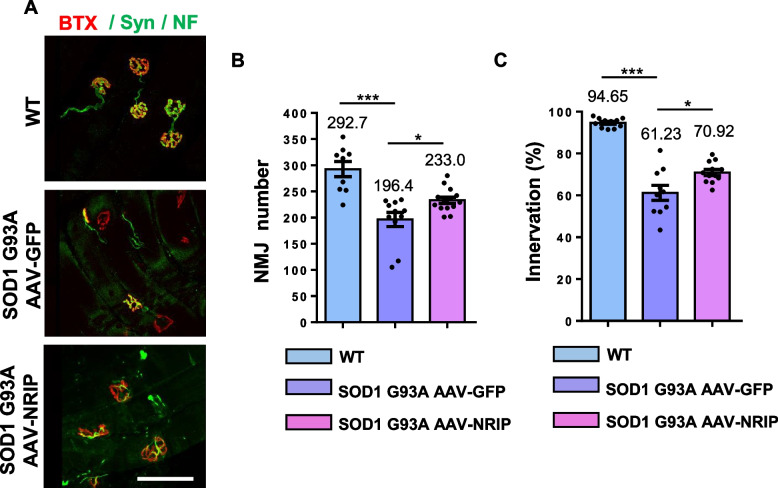
AAV-NRIP gene therapy increases NMJ number and axon terminal innervation at NMJ in SOD1 G93A mice. **A** Immunofluorescence analysis of NMJ and axon terminal innervation at NMJ. GAS muscles were incubated with α-BTX (red) and anti-Syn/NF antibodies (green). Scale bar: 100 μm. **B** Quantification of NMJ number in GAS muscles from WT mice (*N* = 9) and SOD1 G93A mice treated with AAV-GFP (*N* = 11) and AAV-NRIP (*N* = 14) at the age of 120 days. Quantification analysis of NMJ number was counted from the sum of three Sects. (30 μm thickness) of GAS muscles from each mouse. Data are presented as mean ± SEM. ***P = 0.0002 (WT vs. SOD1 G93A + AAV-GFP); *P = 0.0481 (SOD1 G93A + AAV-GFP vs. SOD1 G93A + AAV-NRIP); one-way ANOVA with Tukey’s post hoc test. **C** Axon terminal innervation at NMJ. The co-staining of α-BTX and synaptophysin indicated axon terminal innervation at NMJ of GAS muscles. WT mice (*N* = 11), SOD1 G93A mice infected with AAV-GFP (*N* = 10), and AAV-NRIP (*N* = 12) were analyzed. Quantification analysis of NMJ innervation is defined as the percentage of innervated NMJ to total NMJ number. Data are presented as mean ± SEM. ***P = 0.0002 (WT vs. SOD1 G93A + AAV-GFP); *P = 0.0183 (SOD1 G93A + AAV-GFP vs. SOD1 G93A + AAV-NRIP); one-way ANOVA with Tukey’s post hoc test

NMJ degeneration is reported to occur in the early stages of ALS and persists throughout its progression [32, 46]. NRIP, known for its interaction with AChRα, plays a role in stabilizing NMJ structure [28]. To assess whether AAV-NRIP could reduce NMJ degeneration in SOD1 G93A mice, longitudinal GAS muscle Sects. (30 μm-thick slices) were co-stained with fluoresce-conjugated alpha-bungarotoxin (α-BTX, for AChR labeling), anti-neurofilament (NF, for motor axon), and anti-synaptophysin (Syn, for axon terminals) to analyze NMJ number and axon terminal innervation at NMJ (Fig. 5A). The number of NMJs was reduced in AAV-GFP treated SOD1 G93A mice compared to WT mice (196.4 vs. 292.7, *P* < 0.005; Fig. 5B). AAV-NRIP treatment increased the NMJ number in SOD1 G93A mice compared to AAV-GFP treated mice (233.0 vs. 196.4, *P* < 0.05; Fig. 5B). Additionally, AAV-NRIP also improved axon terminal innervation at NMJ in SOD1 G93A mice compared to AAV-GFP treated mice (70.92% vs. 61.23%, *P* < 0.05; Fig. 5C). In summary, AAV-NRIP gene therapy can effectively improve NMJ degeneration and axon terminal denervation in SOD1 G93A mice.

### Increase in the number of α-motor neurons (α-MNs) and compound muscle action potential (CMAP) by AAV-NRIP gene therapy in ALS model mice

**Fig. 6:**
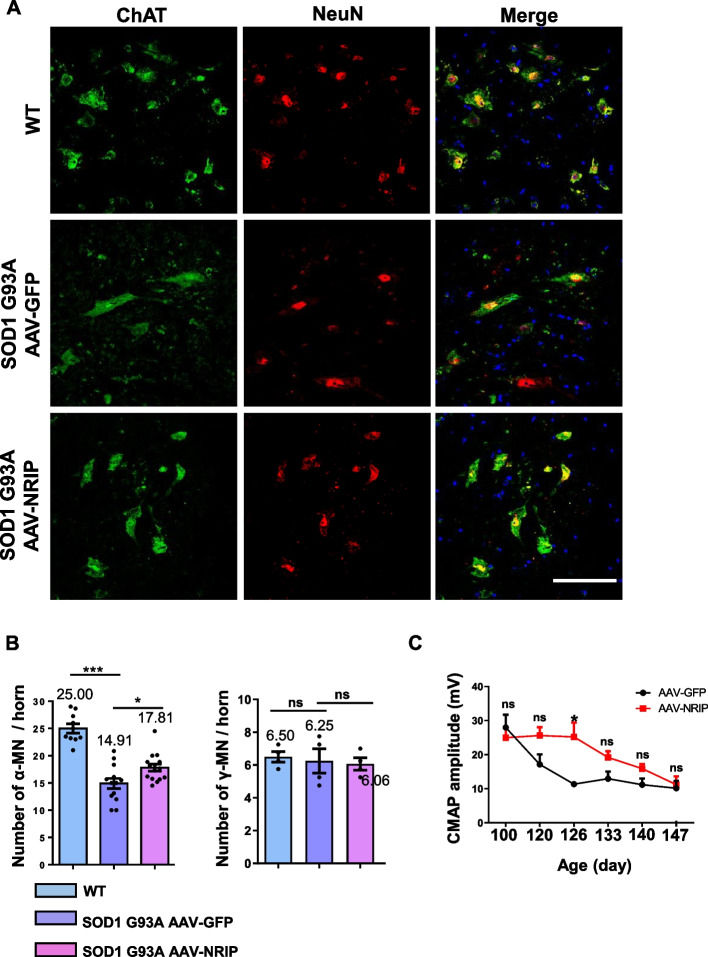
AAV-NRIP gene therapy improves α-MN survival and muscle electrophysiology in SOD1 G93A mice. **A** The therapeutic effect on MN number. Tissue sections from the L3-L5 spinal cord were stained with anti-NeuN and anti-ChAT antibodies to detect α-MN expression and were analyzed by confocal microscopy. Sizes larger than 500 μm^2^ were counted as α-MN. Scale bar: 100 μm. **B** Left panel: Quantification of α-MN numbers per spinal anterior horn in WT mice (*N* = 9) and SOD1 G93A mice treated with AAV-GFP (*N* = 11) and AAV-NRIP (*N* = 14). Data are presented as mean ± SEM. ***P = 0.0003 (WT vs. SOD1 G93A + AAV-GFP); *P = 0.0302 (SOD1 G93A + AAV-GFP vs. SOD1 G93A + AAV-NRIP); one-way ANOVA with Tukey’s post hoc test. Right panel: Quantification of γ-MN numbers per spinal anterior horn in WT mice and SOD1 G93A mice treated with AAV-GFP and AAV-NRIP (*N* = 4 for each group). Data are presented as mean ± SEM. ns, no significance; one-way ANOVA with Tukey’s post hoc test. **C** Compound muscle action potential (CMAP) analysis. The stimulation electrode was placed at the lumbar root, and the active recorder was placed at the GAS muscles of the mice, and the reference recorder was placed at the paw of the hindlimb. Data are presented as mean ± SEM. *P = 0.0147 at the age of 126 days; ns, no significance; one-way ANOVA with Tukey’s post hoc test

ALS is a progressive degenerative disorder affecting motor neurons in the spinal cord, characterized by the selective loss of these neurons [1–3]. The ALS mouse model, SOD1 G93A mice, exhibits a reduced number of α-MNs [31, 47]. In a previous study, our generated muscle-specific NRIP knockout mice displayed a decreased α-MN number, suggesting that muscle NRIP plays a supportive role in α-MN survival [29]. To investigate whether AAV-NRIP gene therapy could rescue motor neuron degeneration, tissue sections of lumbar segments (30 μm-thick sections) were co-stained with choline acetyltransferase (ChAT) and NeuN (Fig. 6A). ChAT/NeuN-double-positive cells with a cell size larger than 500 μm^2^ were defined as α-MNs [48]. The negative or low expression of NeuN in ChAT-positive neurons was described as gamma-motor neurons (γ-MNs) [48, 49]. The quantitative analysis revealed that the total number of ChAT^+^/NeuN^+^ cells in the AAV-GFP treatment group was less than in the AAV-NRIP group (20.06 vs. 24.31, *P* < 0.01; Fig. S6A).The number of ChAT^+^/NeuN^+^ cells larger than 500 µm^2^ (α-MNs) was reduced in AAV-GFP-treated SOD1 G93A mice compared to the AAV-NRIP treatment (14.91 vs. 17.81, *P* < 0.05; Fig. 6B, left panel). There was no significant difference in the number of ChAT^+^/NeuN^+^ cells smaller than 500 µm^2^ between AAV-GFP and AAV-NRIP (Fig. S6B). The number of NeuN^−^/ChAT^+^ cells (γ-MNs) in SOD1 G93A mice was comparable between AAV-NRIP and AAV-GFP treatments (Fig. 6B, right panel). In summary, AAV-NRIP treatment in SOD1 G93A mice could restore α-MNs. Therefore, muscle-delivered AAV-NRIP could effectively rescue the loss of α-MNs.

In conjunction with Figs. 4 and 5B, AAV-NRIP gene therapy demonstrates an increase in α-MN number, NMJ number, and axon terminal innervation. To further assess the impact of AAV-NRIP gene therapy on NMJ transmission, we examined the compound muscle action potential (CMAP) amplitude, an electrophysiological method for evaluating nerve conduction that reflects axon innervation and motor unit number [50]. Both ALS patients and mice typically exhibit lower CMAP amplitudes than normal controls [51, 52]. The CMAP amplitude of hindlimb muscles from SOD1 G93A mice infected with AAV-NRIP or AAV-GFP was measured from 100 days of age (post-infection day 40) to 147 days of age (post-infection day 87). Quantitative analysis revealed that AAV-NRIP increased the CMAP amplitude of SOD1 G93A mice compared to AAV-GFP treatment, with significant differences observed at the ages of 126 days (Fig. 6C). Therefore, AAV-NRIP gene therapy has the potential to enhance NMJ transmission. In summary, AAV-NRIP gene therapy contributes to increased α-MN survival, NMJ number and axon terminal innervation ultimately leading to improved NMJ transmission in SOD1 G93A mice and, consequently, an enhancement in motor activity.

## Discussion

In this study, we observed diminished NRIP expression in the skeletal muscles and spinal cord of SOD1 G93A mice (Fig. 1). These mice are transgenic, expressing the human SOD1 gene with a single amino acid substitution of glycine to alanine at codon 93, driven by its endogenous human SOD1 promoter [12]. This genetic alteration is associated with familial amyotrophic lateral sclerosis (ALS), leading to the development of a paralytic motoneuron disease closely resembling human ALS [11, 53]. NRIP is a multifunctional protein known to directly interact with either calmodulin or α-actinin 2 in the presence of calcium, serving as a calcium sensor and transmitting signals for muscle contraction and maintenance of sarcomere integrity [25, 26, 29]. Additionally, NRIP binds with the acetylcholine receptor (AChR) to stabilize the AChR cluster complex. In the absence of NRIP to reinforce this complex, the efficacy of synaptic transmission and AChR clustering may decline, leading to abnormal neuromuscular junction (NMJ) architecture. This, in turn, results in the retrograde reduction of motor neuron number with impaired motor function, as observed in NRIP knockout mice [28]. The phenotypes observed in muscle-specific NRIP knockout mice resemble ALS, characterized by progressive motor neuron degeneration and NMJ disorders resulting in muscle atrophy. It is reasonable to hypothesize that low NRIP expression in SOD1 G93A mice could be at least one major risk factor contributing to motor neuron degeneration. Our studies showed a decline in NRIP expression in the spinal cord and muscles of SOD1 G93A mice (ALS mouse model). AAV-NRIP-treated SOD1 G93A mice exhibited improved partial motor activities and increased myofiber size. The NRIP expression levels in ALS patients remain unknown. Previously, heart failure and limb-girdle muscular dystrophy (LGMD) patients were found to have lower NRIP RNA expression [26, 54]. Patients with myasthenia gravis who have both anti-AChR and anti-NRIP autoantibodies show worse outcomes than those without the anti-NRIP autoantibody [28], but the NRIP levels in MG patients remain unknown. In the future, AAV-NRIP gene therapy might be a potential tool for treating patients with muscle-related diseases characterized by low NRIP expression.

AAV gene therapy stands out as a clinically prominent platform with significant therapeutic potential. The success of a single intravenous infusion of an AAV vector containing the SMN gene in the treatment of infants with spinal muscular atrophy (SMA) has resulted in longer survival and improved motor function compared to historical cohorts. This achievement underscores the potential of AAV as a delivery vector [55]. In our study, we utilized AAV-NRIP (AAV DJ/8 serotype) as a gene therapy drug to induce NRIP expression in ALS-SOD1G93A mice through intramuscular injection to assess therapeutic efficacy. The results demonstrated the presence of NRIP in muscles (Fig. 2C) following intramuscular delivery of AAV-NRIP and its retrograde transduction to the spinal cord of SOD1 G93A mice (Fig. 2D). In line with prior research, intramuscular injection of AAV (serotype 9) expressing small hairpin RNAs (shRNAs) against mutant SOD1 led to a significant knockdown of mutant SOD1 in muscles and motor neurons [56, 57]. Similarly, intramuscular delivery of AAV-glial cell line-derived neurotrophic factor (AAV-GDNF; serotype 2) was retrogradely transduced from skeletal muscle to the spinal cord, preventing motor neuron degeneration in ALS mice [58]. Consistent with these findings, intramuscular injection of AAV-NRIP (serotype DJ/8) likely could retrogradely transduce NRIP from muscles to the motor neurons of the spinal cord. In summary, the advantages of AAV vectors through intramuscular injection extend beyond muscle expression, encompassing retrograde transduction, which can occur through axon terminal motor endplates to motor neuron cell bodies or via vascular spread to the spinal cord [38].

ALS is a fatal neuromuscular disease characterized by motor neuron degeneration and resulting muscle atrophy [2, 3]. Although the majority of ALS cases are sporadic, approximately 10% are familial. Treatment strategies for ALS can target genetic mutations or involve the introduction of muscle and/or neuron-trophic factors to ameliorate neurodegeneration and muscle atrophy. Among familial genes, about 12% harbor mutations in the SOD1 gene, which encodes the enzyme Cu/Zn superoxide dismutase. SOD1 G93A mice, containing the human SOD1 G93A point mutant, develop paralytic motoneuron disease closely resembling human ALS and are recognized as one ALS mouse model [11, 12]. Therapeutic effects in ALS mouse models are assessed by delaying disease onset and increasing survival [59]. Skeletal muscles and motor neurons reciprocally influence each other, constituting and maintaining functional motor units for motor performance [60]. Hence, assessing the functions of skeletal muscles and motor neurons is crucial for evaluating therapeutic efficacy. In genetic approaches, numerous gene therapy studies utilize RNA interference (RNAi) to eliminate SOD1 expression and prevent disease progression. For instance, intravenous administration of AAV9-SOD1-shRNA into young SOD1 G93A mice can delay disease onset and slow progression. Furthermore, AAV9 delivery after onset markedly slows disease progression and significantly extends survival compared to administration at a younger age [61]. Additionally, it has been documented that intramuscular injections of a lentivirus encoding an shRNA against SOD1 at the age of 7 days can increase lifespan in a SOD mouse model [59]. Thus, the elimination of SOD1 expression using RNAi can effectively improve motor neuron degeneration and muscle atrophy while also increasing survival.

On the other hand, approximately 90% of ALS cases are sporadic, with unclear causative factors. Muscle atrophy and motor neuron loss are common features in both sporadic and familial ALS [30]. Therefore, the component genes of the neuromuscular junction (NMJ) or muscle and/or neuron trophic factors become potential therapeutic targets for intervention. Given that NRIP is a component of NMJ through acetylcholine receptor (AChR) binding [28], we evaluated AAV-NRIP as a potential drug for ALS-SOD1G93A. AAV-NRIP gene therapy was administered at the age of 60 days before the onset of disease [21] and pathological changes were observed at the age of 120 days (60 days after AAV-NRIP administration). AAV-NRIP gene therapy in SOD1 G93A mice revealed enlarged myofiber size (Fig. 4), increased NMJ number and axon terminal innervation (Fig. 5), enhanced α-MN survival (Fig. 6B), and elevated NMJ transmission (Fig. 6C), leading to improvements in motor functions such as movement distance (Fig. 3A) and rearing frequencies (Fig. 3B). Regarding lifespan, AAV-NRIP did not have a significant effect on the survival of SOD1 G93A mice (Fig. S5C). Currently, components of the NMJ have been explored as therapeutic drugs for ALS-SOD G93A. For instance, Dok7, functioning in the agrin-Lrp4-MuSK pathway of NMJ, enhances MuSK autophosphorylation involved in acetylcholine receptor (AChR) phosphorylation to stabilize AChR complex formation. AAV-Dok7 gene therapy suppresses motor nerve terminal degeneration at NMJs, mitigates muscle atrophy, and enhances motor activity and lifespan through tail vein injection at age 90 days (symptomatic stage) in the SOD1 G93A-ALS mouse model. Another example is MuSK, a receptor tyrosine kinase and NMJ component in the agrin-Lrp4-MuSK pathway. MuSK transgenic mice crossed with SOD1G93A mice show increased MuSK expression, delaying onset and reducing muscle denervation extent, thereby improving motor function without altering survival [23]. These findings suggest that NMJ components could be potential pharmacological drugs to improve muscle and motor functions, akin to the effects observed with AAV-Dok7 gene therapy or transgenic MuSK. Therefore, NRIP, as a component of the AChR complex [28], demonstrates therapeutic efficacy through AAV-NRIP gene therapy, mirroring the effects observed with AAV-Dok7 gene therapy or transgenic MuSK.

NRIP is a multifunctional protein that not only acts as an acetylcholine receptor (AChR) binding protein but also functions as a binding protein for calmodulin and α-actinin 2. These interactions are involved in muscle contraction, sarcomere maintenance, and the regulation of mitochondria [25, 26, 29]. The diverse roles of NRIP in these processes make it comparable to muscle/neuron trophic factors. Muscle/neurotrophic factors have been explored as therapeutic drugs for ALS mouse models. For instance, intramuscular injection of AAV-IGF1 inhibits skeletal muscle atrophy and enhances motor neuron survival in SOD1 G93A mice [19]. Adenovirus-mediated myogenin gene transfer into hindlimb muscles improves neuromuscular junction (NMJ) innervation in SOD1 G93A mice and enhances motor neuron survival [31]. Activating transcription factor 3 (ATF3), acting as a neuron growth factor, increases motor neuron survival and maintains axonal connections with muscles by promoting axonal sprouting when overexpressed in motor neurons in an ALS mouse model through ATF3 transgenic mice expressing the ATF3 transgene under the control of the thy1.2 promoter [62]. Additionally, mitochondrial-targeted therapeutic drugs have been proposed as targets for toxicity in ALS [63]. Cyclophilin D, which decreases the capacity of spinal cord mitochondria, shows potential when eliminated in cyclophilin D-null mice crossed with SOD1 mice, leading to improved mitochondrial ATP synthesis and rescuing motor neuron death and muscle paralysis [64]. The muscle fiber type profile is primarily determined by nerve activity and can change in response to either neural or hormonal influences [65]. In our previous study, NRIP was shown to activate slow myosin expression [25]. Here, we only examined the soleus muscles, which contain a higher proportion of slow myosin. Consistent with our previous report [25], our results showed that NRIP-positive cells increased slow myosin expression compared to NRIP-negative cells (Fig. 4F), and AAV-NRIP treated SOD1 G93A mice exhibited an increased percentage of slow myosin fibers (Fig. 4E). A question arises as to whether NRIP can switch glycolytic fibers (fast myosin) to oxidative fibers (slow myosin); this will be interesting for further investigation.

As reported, both the fast-twitch and slow-twitch muscles of SOD1 G93A mice show reduced myofiber diameter at 120 days of age compared to age-matched wild-type mice [66]. Here, we found that the overall myofiber sizes could be increased by AAV-NRIP treatment in SOD1 G93A mice (Fig. 4B and C). The enlarged myofiber sizes may include both slow and fast myofibers. Whether the fast myofibers are affected by AAV-NRIP treatment needs to be determined in future studies. In general, slow myosin functions for maintaining posture, while fast myosin provides muscle mechanical power. However, in myotonic dystrophy type 1, oxidative fibers (slow) tend to be most prominent in muscles, which is suggested to be one reason for causing myotonia [67]. The therapeutic doses of AAV-NRIP will be further evaluated. In summary, NRIP's interactions with various proteins such as AChR, calmodulin, and α-actinin 2 influence diverse muscle functions and retrogradely control motor neuron growth, all of which may contribute to the therapeutic efficacy of AAV-NRIP gene therapy in ALS mouse models.

The primary advantage of AAV intramuscular injection lies in its ability for retrograde transduction to motor neurons. However, this approach faces challenges related to immune responses directed against viral capsid proteins or the targeted gene. These side effects are, in part, a consequence of tissue injury and the concentrated antigen load [68]. For instance, intramuscular injection of AAV-microdystrophin into dystrophic dogs, driven by the cytomegalovirus (CMV) promoter, resulted in a significant immune response to both the transgene protein [69] and AAV capsids [70]. Another concern is the potential for targeted gene expression in non-muscle tissues, which may elicit an immune response [71]. To mitigate immune responses to the targeted protein, muscle-specific regulatory cassettes that restrict expression to skeletal and cardiac muscles can be employed [72, 73]. Alternatively, alternative injection methods, such as intravascular delivery, have been explored to minimize immune responses [71]. Although we did not investigate the immune response to NRIP in this study, it remains a subject for future research. Moreover, it has been reported that axonal transport is selectively impaired in SOD1 G93A mice, which could reduce retrograde transport from muscle to the spinal cord [74]. This could also result in the limited therapeutic effect of AAV-NRIP on motor performance and survival of SOD1 G93A mice. A single injection of AAV9 expressing green fluorescent protein (AAV9-GFP, 9 × 10^10^ vg per mouse) into the adult mouse gastrocnemius induces major transduction at the injection site and partial transduction of the contralateral (noninjected) gastrocnemius, brain, and peripheral organs through the bloodstream [38]. IM injection of AAV8 expressing firefly luciferase (AAV8-ffLuc, 1 × 10^10^ vg per mouse) into mouse gastrocnemius muscles also results in major expression of ffLuc at the injection site and in the liver [75]. Hence, IM injection of AAV can spread to other tissues through the bloodstream but shows lower expression levels than at the injection site. Here, we used IM injection of AAV-NRIP with a similar dose of 8 × 10^10^ vg per mouse, which was sufficient to result in NRIP expression in the injected skeletal muscles and retrograde transduction to the spinal cord. However, the survival of SOD1 G93A mice was not improved at this dose of AAV-NRIP, possibly due to an insufficient level of NRIP expression in the diaphragm (this needs to be further investigated) to support the respiratory function in SOD1 G93A mice.

## Conclusions

Low NRIP expression was observed in the skeletal muscles and spinal cord of SOD1 G93A mice. Intramuscular injection of AAV-NRIP successfully restored NRIP expression in skeletal muscles and retrogradely transduced it in the spinal cord. Importantly, AAV-NRIP gene therapy in ALS mice rescued muscle atrophy, increased axon terminal innervation at the neuromuscular junction (NMJ), elevated NMJ number, and expanded α-motor neuron (α-MN) survival. These improvements led to an escalation in NMJ transmission, ultimately contributing to the enhancement of motor activity. The NMJ and motor neuron degeneration as well as muscle atrophy occur in both sporadic and familial ALS [32]. Therefore, AAV-NRIP may not only improve SOD1 G93A mice but also potentially alleviate other types of ALS. Future efforts will focus on optimizing the injection route, timing of therapeutic administration, and employing tissue-specific promoters to drive NRIP gene expression, aiming to enhance therapeutic effects while minimizing potential side effects. In conclusion, NRIP emerges as a promising candidate for gene therapy in ALS.

## Materials and methods

### Animal study

The SOD1 G93A [B6SJL-Tg (SOD1*G93A)1Gur/J] mice were generously provided by Dr. Jun-An Chen (Institute of Molecular Biology, Academia Sinica, Taiwan) [47]. The SOD1 G93A transgene contains a mutant human *SOD1* gene, in which a single amino acid substitution of glycine to alanine occurs at codon 93, under the control of the human *SOD1* promoter. Wild-type (WT) mice were obtained from non-transgenic SOD1 G93A littermates. Tail DNA of transgenic offspring was extracted using DirectPCR (Viagen) and subjected to genotyping by polymerase chain reaction (PCR) amplification. The primer sequences for the transgene were as follows: forward CATCAGCCCTAATCCATCTGA, reverse CGCGACTAACAATCAAAGTGA. Transgene copy numbers were confirmed using real-time PCR by determining the difference in cycle threshold (ΔCt) between a reference gene (mouse *apob*) and the transgene (human *SOD1*), following the recommendations of The Jackson Laboratory. We used both male and female mice from SOD1 G93A mice and wild-type littermates for our experiments. Experimental mice were housed in the animal center under a 12-h light/dark cycle with free access to food and water.

### AAV-NRIP generation and injection

The PCR-amplified DNA fragments of Flag-tagged NRIP were subcloned into the AAV-MCS vector. To generate AAV-NRIP and AAV-GFP, HEK293T cells were co-transfected with pAAV-MCS-NRIP or pAAV-GFP, pAAV-DJ/8 (Cell Biolabs, INC), and the adenovirus helper plasmid pHelper using calcium phosphate transfection for 72 h. AAV particles were purified by CsCl density-gradient ultracentrifugation [76]. CsCl was removed by dialysis with a dialysis cassette (Slide-A-Lyzer dialysis cassettes, Thermo; MWCO 10 K) in dialysis buffer (350 mM NaCl with 5% sorbitol in PBS at 4 °C). The purified AAV-NRIP or AAV-GFP (1 × 10^10^ vg / each injection) were then injected into the bilateral hind limbs (gastrocnemius and tibialis anterior muscles) and forelimbs (triceps and biceps) of 60-day-old SOD1 G93A mice. Each mouse received eight injections, totaling 8 × 10^10^ vg / mouse. Therapeutic effects on NMJ innervation and α-MN were analyzed at 120 days of age. The evaluation of locomotor activity was performed from 100 days of age to 147 days of age, and the survival rate was analyzed from the day of birth to the end stage. The efficacy of grip force and rotarod tests were performed at 120 days of age.

### Behavioral test

For locomotor activity, mice were allowed to move freely within a clean, opaque acrylic box (40 × 40 × 40 cm^3^) for 10 min. The movement of the mice was tracked using EthoVision Video Tracking Software (Noldus). The total distance moved (total distance traveled by mice in the box) and the rearing frequency (frequency at which mice stand on their hindlimbs in the box) were measured during the test. For the rotarod assay, mice were placed on the 3-cm diameter cylinder of the rotarod apparatus (Singa RT-01) and trained at a constant rotating speed of 5 rpm for 1 min three times before testing (10 min breaks were provided in each training interval). The mice were given a 30-min break between the training and test phases. In the experimental phase, mice were tested at a constant rotating speed of 10 rpm for 5 min three times (15 min breaks were provided in each testing interval), and the time at which each mouse fell off the rod in the last test was recorded [25]. For the grip force test, grip strength was measured using the grip strength meter five times. The forelimbs of mice were placed on a horizontal bar, and their tails were pulled until they released the bar to measure the peak grip strength.

### Compound muscle action potential (CMAP) amplitude analysis

At the age of 100 days, mice were anesthetized with Zoletil (20 mg/kg, intraperitoneally), and the hair on the right hindlimb and lower back was shaved using a depilatory cream before recording. The stimulation electrode was placed at the lumbar root, the active recorder was positioned at the gastrocnemius muscle of the mice, and the reference recorder was located at the paw of the hindlimb.

### Survival analysis

We used the Kaplan–Meier curve with a log-rank test to compare survival rate for each group. Survival in SOD1 G93A mice was recorded from the day of virus treatment to the end stage. The end stage of SOD1 G93A mice was defined as the day when mice were unable to right themselves within 15 s [40]. The mice were sacrificed at the end stage.

### Immunofluorescence analysis

For α-motor neuron staining, the spinal cord was dissected and rapidly frozen with O.C.T. compound (Sakura) at liquid nitrogen. The tissue blocks were then sectioned into 30 μm sections and fixed in 4% paraformaldehyde (PFA) for 3 min. Following that, sections were blocked in 5% bovine serum albumin (BSA) and 1% horse serum (HS) for 1.5 h at room temperature and incubated with the anti-NeuN (Millipore, MABN140, 1:500) and anti-ChAT (Millipore, AB144P, 1:250) overnight at 4 °C. After primary antibodies reaction, sections were washed three times in phosphate buffer saline (PBS) for 10 min each and underwent secondary incubation (Cy3-conjugated goat anti-rabbit or 488-conjugated goat anti-goat, 1:1000, Jackson ImmunoResearch Laboratories) for 1 h at room temperature. Finally, sections were washed in PBS for 10 min three times in dark and mounted with DAPI Fluoromount-G (SouthernBiotech). The immunofluorescence signals were visualized and captured using by Zeiss Axioskop 40 Optical Microscope using the AxioCam 702 camera and Zeiss Zen blue software. Ventral horn cells with NeuN and ChAT double-positive signal with cell size larger than 500 μm^2^ were identified as α-motor neurons.

For neuromuscular junction staining, the gastrocnemius (GAS) muscles were fixed in 2% paraformaldehyde (PFA) and then embedded in O.C.T. compound (Sakura). The 30-μm-thick GAS frozen sections were blocked in blocking buffer containing 0.2% Triton X-100 and 2% BSA in PBS for 1 h at room temperature and incubated with the anti-neurofilament (anti-NF, Abcam, ab8135, 1:500) and anti-synaptophysin (anti-Syn, Abcam, ab32127, 1:250) in blocking buffer overnight at 4 °C. After washing, the sections were incubated with secondary antibody (488-conjugated goat anti-rabbit, 1:1000, Jackson ImmunoResearch Laboratories) and Alexa-594-conjugated α-bungarotoxin (α-BTX, Life technologies, B13423, 1:1000) for 2 h. The immunofluorescence images were visualized and captured by Carl Zeiss LSM880 confocal microscope and Zeiss Zen black software. To quantify the number of NMJ, gastrocnemius muscles from each mouse (WT, *N* = 9 mice; SOD1 G93A with AAV-GFP, *N* = 11 mice; SOD1 G93A with AAV-NRIP, *N* = 14 mice) had neuromuscular junctions with a pretzel-shaped morphology, which were positive for α-bungarotoxin (α-BTX) binding to the acetylcholine receptor (AChR), quantified on three nonadjacent sections spaced at least 100 μm apart [62]. The overlap of α-BTX, anti-NF and anti-Syn was defined as axonal innervation; conversely, α-BTX only considered as axonal denervation.

For myofiber CSA and slow myosin staining, the 10-μm-thick cross-sections of GAS muscles were incubated with anti-Flag (Abcam, ab1162, 1:200) and anti-slow myosin (Abcam, ab11083, 1:500) primary antibodies for Flag-NRIP expression in slow myosin-positive myofibers; or incubated with anti-laminin (Novus, NB300-144, 1:200) for myofiber boundaries staining. The immunofluorescence images were visualized and captured by Carl Zeiss LSM880 confocal microscope and Zeiss Zen black software.

### Western blot

Spinal cord and muscle tissues were harvested and immediately stored in liquid nitrogen. Tissues lysates were extracted by RIPA lysis buffer (150 mM NaCl, 50 mM Tris and 1% NP-40) with 1% protease inhibitor and 0.1% SDS for 30 min incubation on ice. Proteins were separated on 10% sodium dodecyl sulfate polyacrylamide gel electrophoresis (SDS-PAGE) and transferred to polyvinylidene fluoride (PVDF) membranes (Millipore), then were blocked in 5% BSA for 1 h at room temperature. Then, membranes were incubated with the primary antibodies anti-NRIP (Novus, NBP1-30,075, 1:2000), anti-GFP (SantaCruz, sc-9996, 1:5000), anti-Flag (Sigma, F3165, 1:2000) and anti-GAPDH (AbFrontier, LF-PA0212, 1:10,000) diluted in blocking buffer at 4 °C overnight and followed by incubation with HRP-conjugated secondary antibody for 1 h at room temperature. Target protein expression was detected by using an ECL western blot detection system (GE Healthcare Life Sciences, Chicago, IL, USA).

### Statistical analysis

All statistical data were analyzed using Prism (GraphPad Software). Data are reported as the mean ± SEM. *P*‐values were determined using Student's t‐test for comparing two groups, or by one-way ANOVA for comparing more than two groups. *P* < 0.05 was considered statistically significant.

### Supplementary Information

Supplementary Material 1.

## Data Availability

No datasets were generated or analysed during the current study.

## Abbreviations

AAV: Adeno-associated virus
AChR: Acetylcholine receptor
ACTN2: Alpha-actinin 2
ALS: Amyotrophic lateral sclerosis
AR: Androgen receptor
ASO: Antisense oligonucleotide
ATF3: Activating transcription factor 3
ATP: Adenosine triphosphate
α-BTX: Alpha-bungarotoxin
Ca^2+^: Calcium
CaM: Calmodulin
ChAT: Choline acetyltransferase
CMAP: Compound muscle action potential
CMV: Cytomegalovirus
CSA: Cross-sectional area
DCAF6: DDB1 and CUL4 associated factor 6
Dok-7: Docking protein-7
FDA: Food and Drug Administration
GAS: Gastrocnemius
GDNF: Glial cell line-derived neurotrophic factor
GFP: Green fluorescent protein
IGF1: Insulin-like growth factor 1
IQ: Isoleucine and glutamine
IQWD1: IQ motif and WD repeat-containing protein 1
LGMD: Limb-girdle muscle dystrophy
Lrp4: Lipoprotein receptor related protein 4
MG: Myasthenia gravis
MLP: Muscle LIM protein
MN: Motor neuron
MND: Motor neuron disease
MuSK: Muscle-specific tyrosine kinase receptor
NeuN: Neuronal nuclei
NF: Neurofilament
NMJ: Neuromuscular junction
NRIP: Nuclear receptor interaction protein
NRIP gKO: Global NRIP knockout
NRIP cKO: Muscle-specific NRIP knockout
PMA: Progressive muscular atrophy
shRNA: Small hairpin RNA
RNAi: RNA interference
SMA: Spinal muscular atrophy
SOD1: Superoxide dismutase type 1
Syn: Synaptophysin
TA: Tibialis anterior
WT: Wildtype
