# Supplementary material for: AAV-NRIP gene therapy ameliorates motor neuron degeneration and muscle atrophy in ALS model mice
Source: Skelet Muscle. 2024 Jul 24;14:17. doi: 10.1186/s13395-024-00349-z (PMC11267858; doi:10.1186/s13395-024-00349-z)

Supplementary Information

Supplementary Figure Legends

Fig. S1 NRIP expression in the spinal cord and skeletal muscles at 4 and 6 weeks of age in WT and SOD1 G93A mice. (**A**) NRIP expression in the spinal cord at 4 weeks of age in WT and SOD1 G93A mice. Total proteins from L3-L5 spinal cord were subjected to Western blot (WB) analysis for NRIP expression, with GAPDH serving as the loading control. The right panel shows the quantification of NRIP expression conducted through densitometry analysis. (**B**) NRIP expression in the gastrocnemius (GAS) muscles at 4 weeks of age in WT and SOD1 G93A mice. The right panel illustrates the quantification. (**C**) NRIP expression in the tibialis anterior (TA) muscles at 4 weeks of age in WT and SOD1 G93A mice. The right panel displays the quantification. (**D**) NRIP expression in the spinal cord at 6 weeks of age in WT and SOD1 G93A mice. The right panel represents the quantification of NRIP expression. (**E**) NRIP expression in the gastrocnemius (GAS) muscles at 6 weeks of age in WT and SOD1 G93A mice. The right panel illustrates the quantification. (**F**) NRIP expression in the tibialis anterior (TA) muscles at 6 weeks of age in WT and SOD1 G93A mice. The right panel shows the quantification. Each group consisted of N = 3 mice for both WT and SOD1 G93A. Data are presented as mean ± SEM. Statistical analysis was conducted using the Student t-test. *$P < 0.05$ and **$P < 0.01$.

Fig. S2 Immunofluorescence analysis of AAV-GFP expression in the spinal cord of WT mice via intramuscular injection. The analysis was conducted on both control WT mice (without AAV-GFP treatment) and WT mice administered with AAV-GFP through intramuscular injection. The spinal cord was dissected into cervical, thoracic, and lumbar segments. Each segment was co-stained with anti-GFP (green, indicating GFP expression) and anti-NeuN (red, a neuron marker) antibodies. DAPI was used as the nuclear stain. Scale bars: 100 μm.

Fig. S3 Transgene copy number determination in SOD1 G93A mice treated with AAV-NRIP and AAV-GFP. The transgene copy number in SOD1 G93A mice treated with AAV-NRIP and AAV-GFP was determined by real-time PCR. The ΔCt (cycle threshold) of transgene copy number was assessed by comparing a reference gene (mouse *apob*) with the transgene (human *SOD1)*. The AAV-GFP group comprised 3 mice, and the AAV-NRIP group comprised 4 mice. Data are presented as mean ± SEM. Statistical analysis was performed using the Student t-test. ns, no significance.

Fig. S4 Total distance moved and rearing frequency of SOD1 G93A mice with AAV-GFP or AAV-NRIP treatment in males and females. (**A**) The total distance moved by SOD1 G93A mice with AAV-GFP or AAV-NRIP treatment was recorded from 100 to 147 days using EthoVision Video Tracking Software (Noldus) during a 10-minute test. The total distance traveled in the box was measured. In the male group, data are presented as mean ± SEM. **P* =0.045 at the age of 126 days; ns, no significance; one-way ANOVA with Tukey's post hoc test. In the female group, data are presented as mean ± SEM. **P* =0.0496 at the age of 120 days; ns, no significance; one-way ANOVA with Tukey's post hoc test. (**B**) Rearing frequency, indicating the frequency of mice standing on their hindlimbs in the box, was recorded from 100 to 147 days. In the male group, data are presented as mean ± SEM. **P* =0.0166 at the age of 126 days; ns, no significance; one-way ANOVA with Tukey's post hoc test. In the female group, data are presented as mean ± SEM. **P* =0.0381 at the age of 120 days; **P* =0.0429 at the age of 133 days; ns, no significance; one-way ANOVA with Tukey's post hoc test.

Fig. S5 Therapeutic effects of AAV-NRIP on rotarod performance and grip force via intramuscular injection in SOD1 G93A mice. (**A**) Evaluation of AAV-NRIP efficacy in rotarod performance of SOD1 G93A mice at the age of 120 days. Mice were placed on a rotating rod set at 20 rpm, and the time until falling was recorded. N=6 for each group. (**B**) Analysis of grip force. Forelimb strength was measured five times for each mouse at the age of 120 days. N=6 for each group. Data are presented as mean ± SEM. Statistical analysis was conducted using the Student t-test. ns, no significance. (**C**) Kaplan-Meier survival curves comparing AAV-NRIP-treated and AAV-GFP-treated SOD1 G93A mice. Statistical differences were analyzed using the Logrank test. ns, no significance.

Fig. S6 AAV-NRIP gene therapy improves ChAT and NeuN co-positive MN survival in SOD1 G93A mice. (**A**) Quantification of total $ChAT^+/NeuN^+$ MN numbers per spinal anterior horn in WT mice (N=4) and SOD1 G93A mice treated with AAV-GFP (N=4) and AAV-NRIP (N=4). Data are presented as mean ± SEM. ***P* =0.0026 (WT vs. SOD1 G93A+AAV-GFP); ***P* =0.0036 (SOD1 G93A+AAV-GFP vs. SOD1 G93A+AAV-NRIP); one-way ANOVA with Tukey's post hoc test. (**B**) Quantification of $ChAT^+/NeuN^+$ MN numbers with a cell size smaller than 500 μm^2 per spinal anterior horn in WT mice (N=4) and SOD1 G93A mice treated with AAV-GFP (N=4) and AAV-NRIP (N=4). Data are presented as mean ± SEM. Statistical analysis was conducted using the one-way ANOVA with Tukey's post hoc test. ns, no significance.

Figure S1

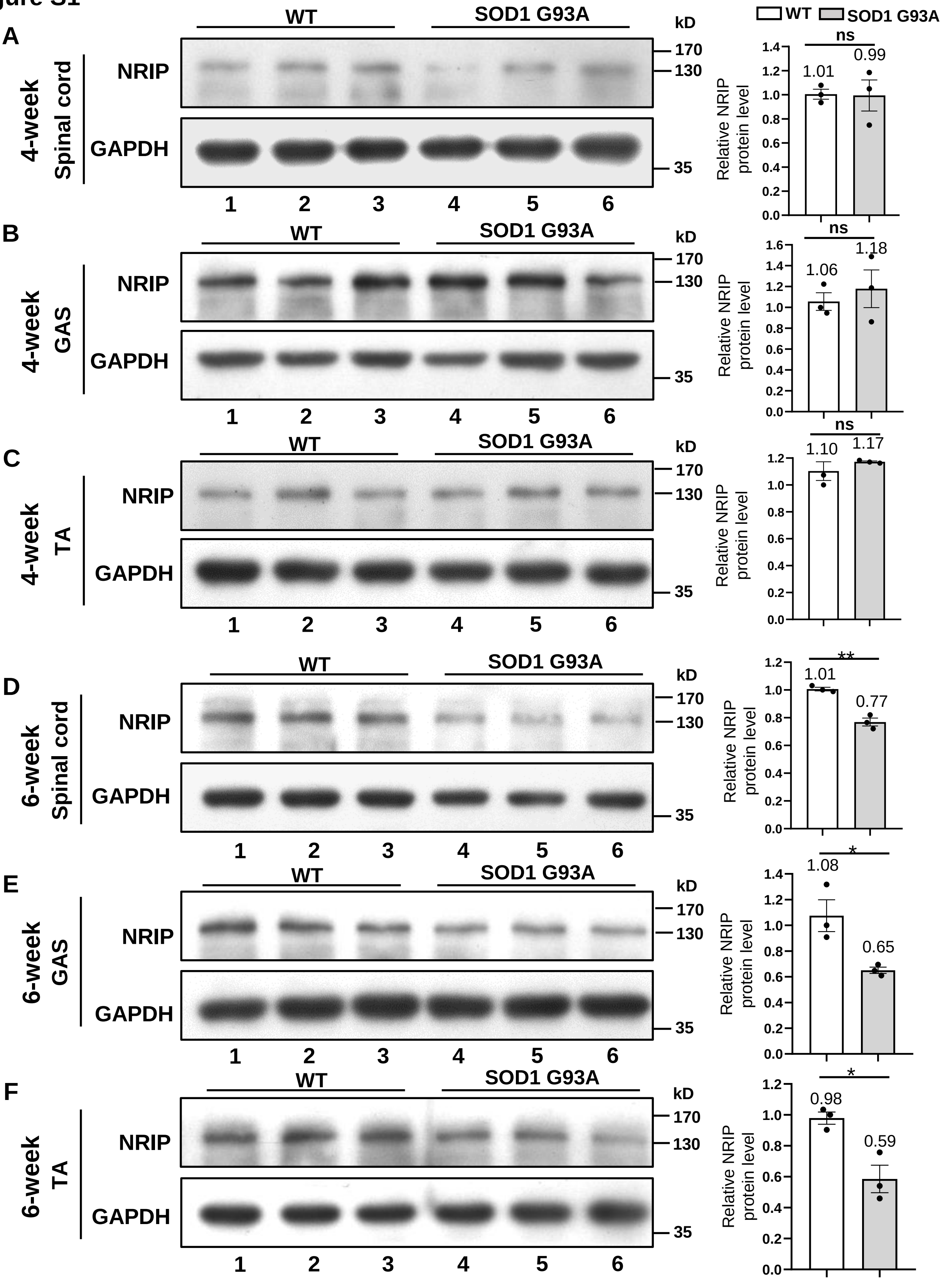
A
B
C
D
E
F
WT
SOD1 G93A
kD
170
130
35
NRIP
GAPDH
1
2
3
4
5
6
4-week
Spinal cord
GAS
TA
6-week
Relative NRIP protein level
ns
1.01
0.99
1.06
1.18
1.10
1.17
**
0.77
*
1.08
0.65
0.98
0.59

Figure S2

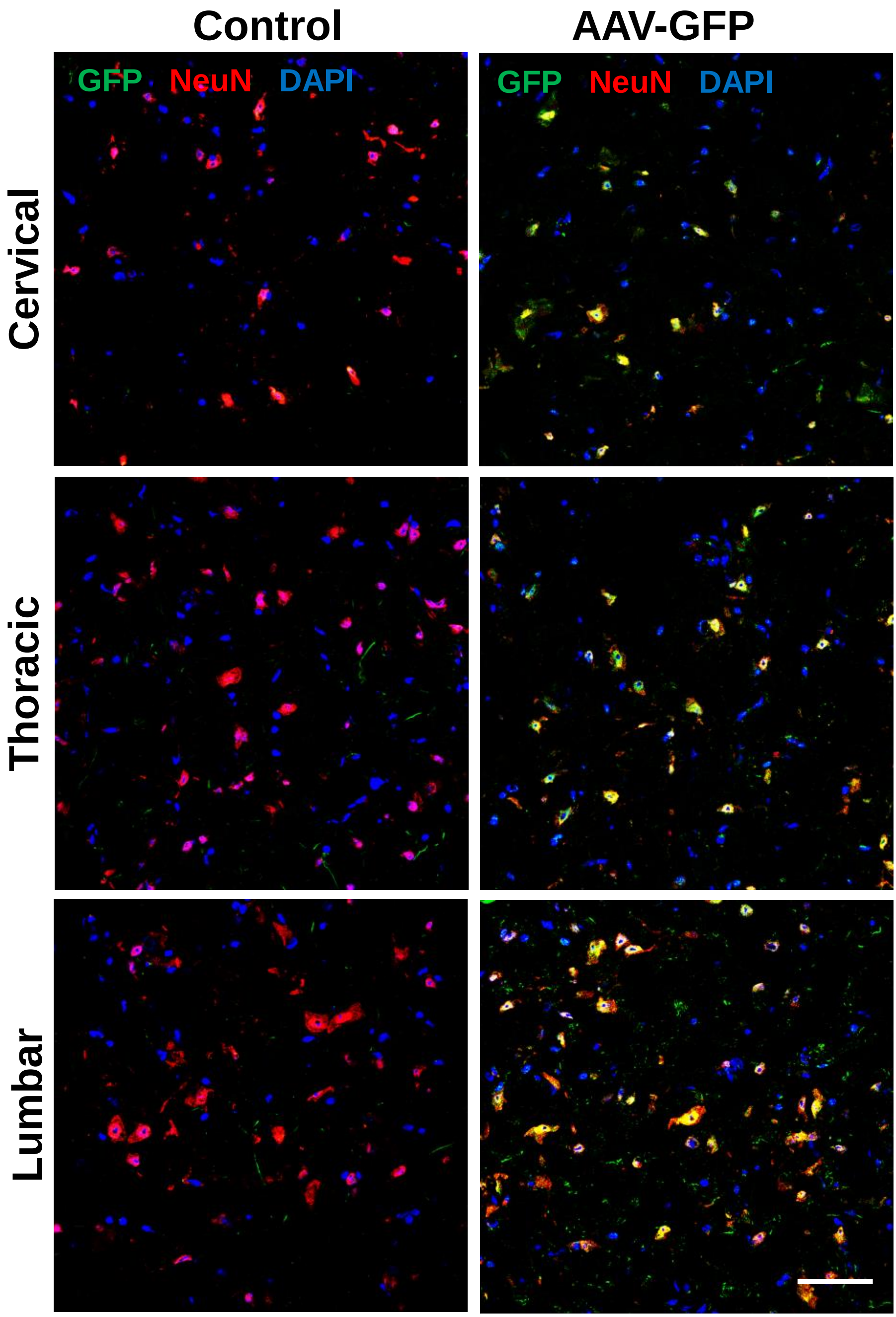

Control
AAV-GFP
GFP NeuN DAPI
GFP NeuN DAPI
Cervical
Thoracic
Lumbar

Figure S3

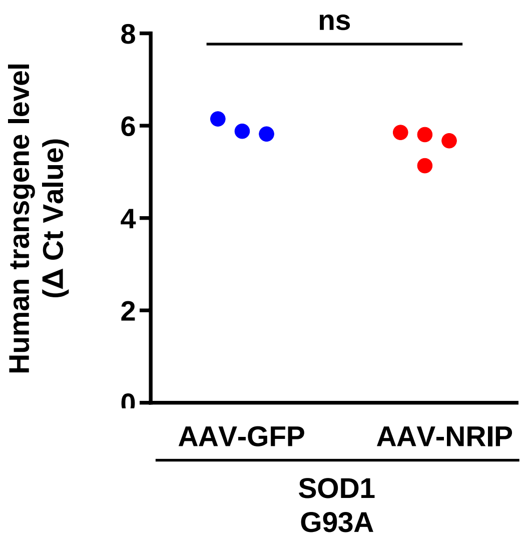
ns
Human transgene level
(Δ Ct Value)
8
6
4
2
0
AAV-GFP
AAV-NRIP
SOD1
G93A

Figure S4

A

Male **Female**

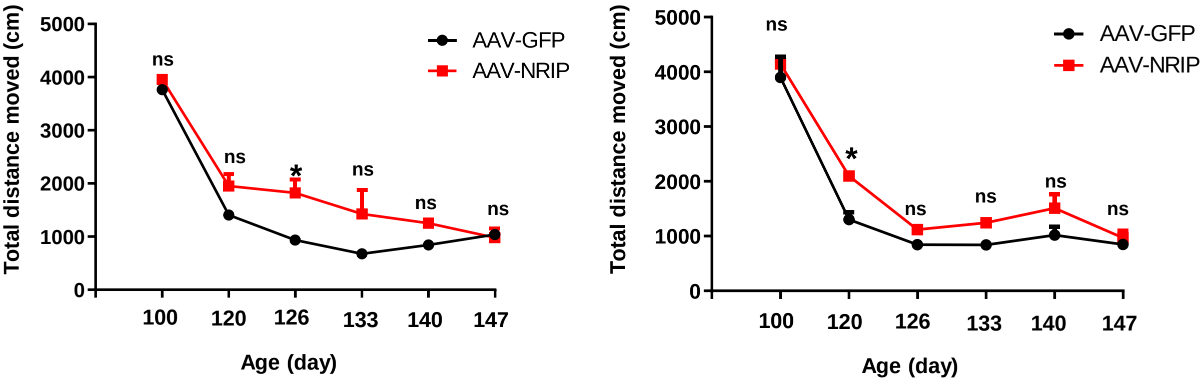

B

Male **Female**

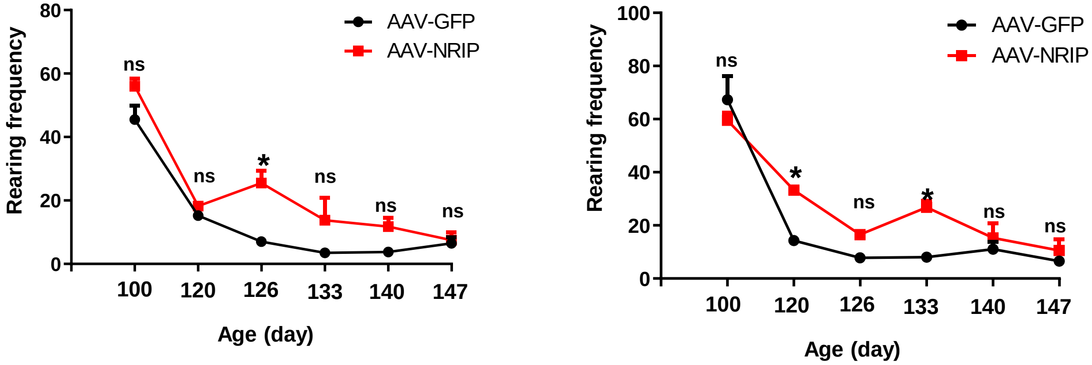

Figure S5

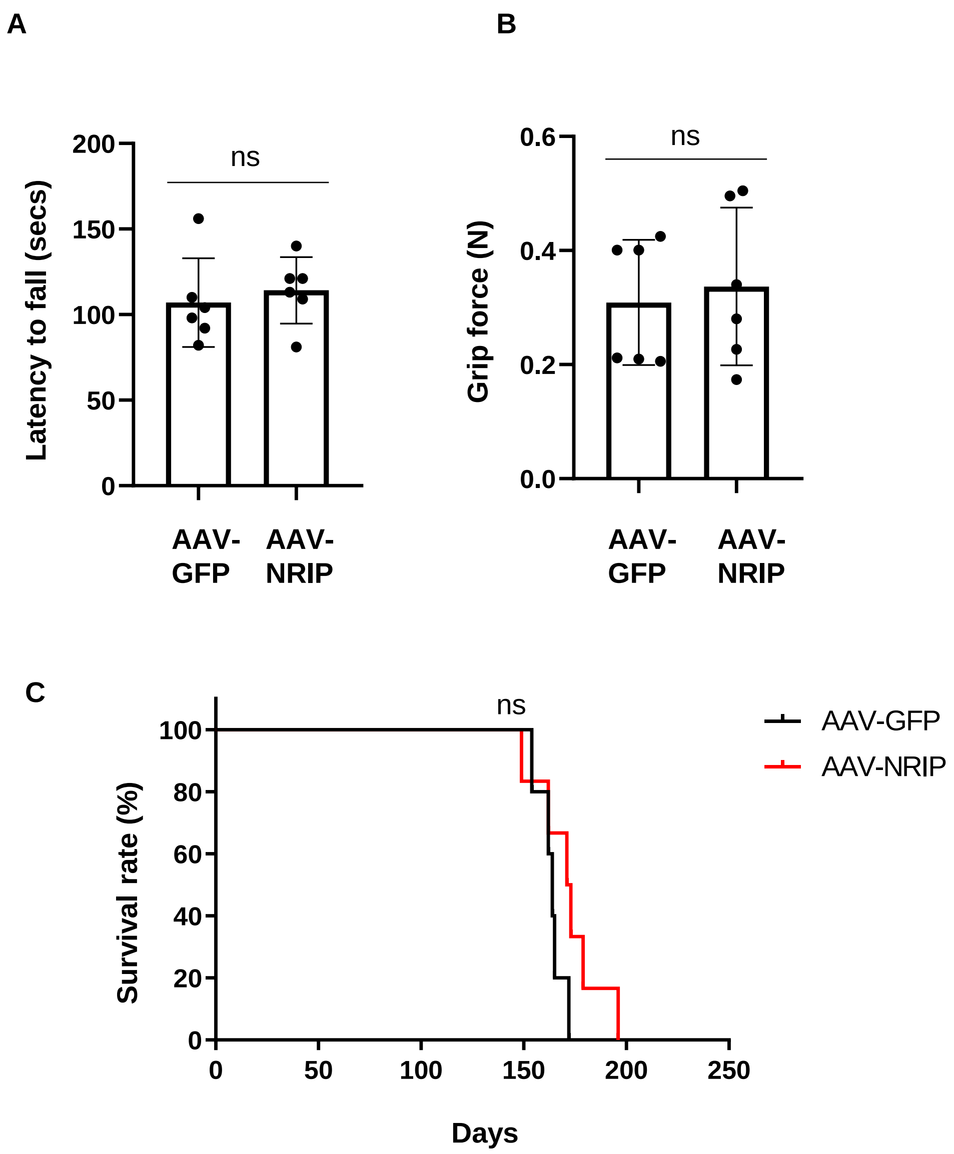
A
B
200
150
100
50
0
Latency to fall (secs)
ns
AAV-GFP
AAV-NRIP
0.6
0.4
0.2
0.0
Grip force (N)
ns
AAV-GFP
AAV-NRIP
C
ns
100
80
60
40
20
0
Survival rate (%)
0
50
100
150
200
250
Days
AAV-GFP
AAV-NRIP

Figure S6

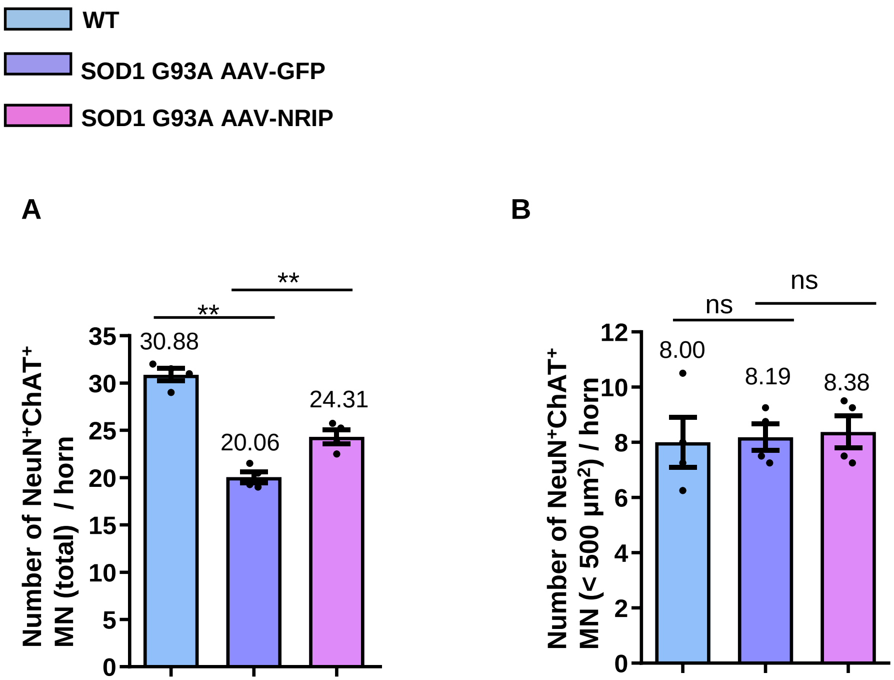

WT
SOD1 G93A AAV-GFP
SOD1 G93A AAV-NRIP
A
**
**
30.88
20.06
24.31
Number of NeuN+ChAT+ MN (total) / horn
0
5
10
15
20
25
30
35
B
ns
ns
8.00
8.19
8.38
Number of NeuN+ChAT+ MN (< 500 μm2) / horn
0
2
4
6
8
10
12

Supplementary Information

Grayscale images of Fig.1D, Fig. 2C, Fig. 2D, Fig. 4A, Fig. 4D, Fig. 5A,Fig. 6A and Fig. S2

**Figure 1D
Grayscale**

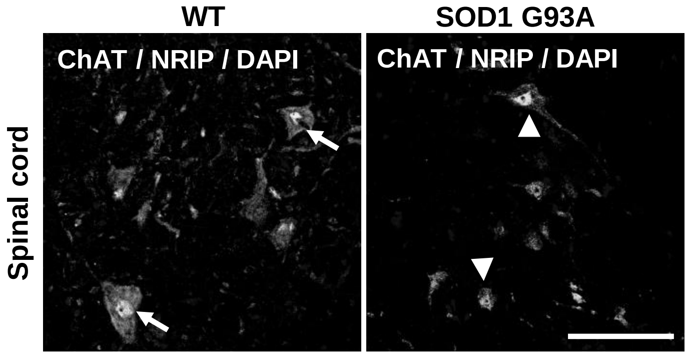

**Figure 2C
Grayscale**

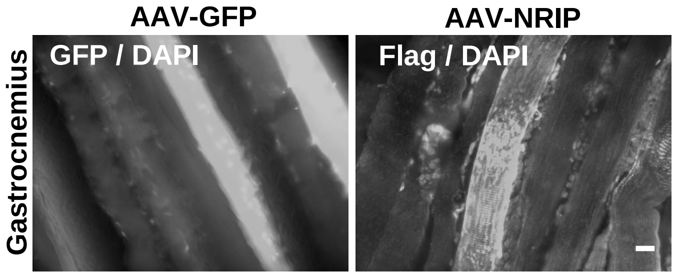

**Figure 2D
Grayscale**

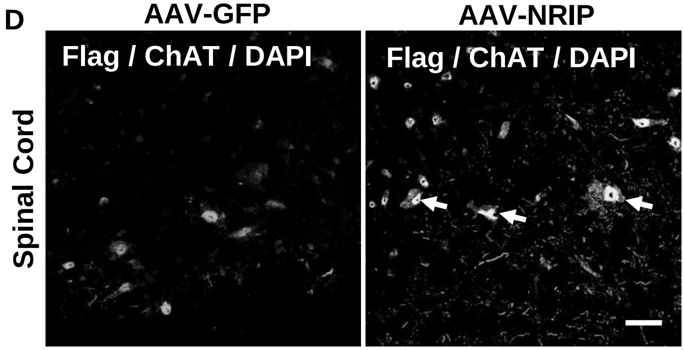

Figure 4A
Grayscale

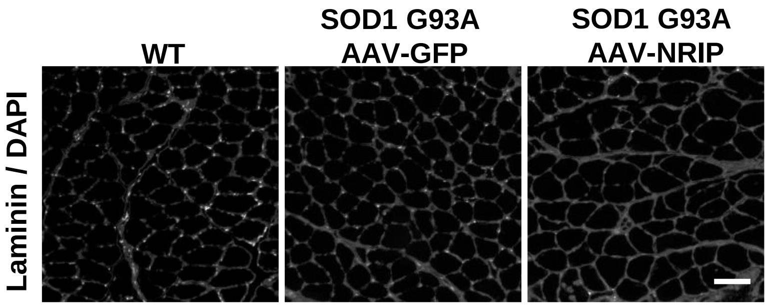

Figure 4D
Grayscale

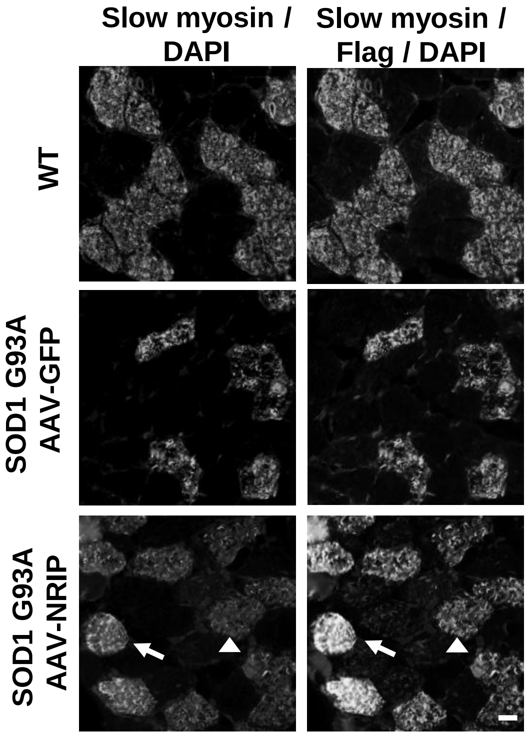

Figure 5A
Grayscale

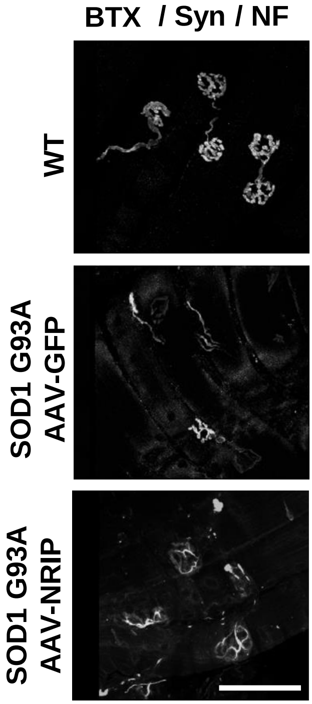

Figure 6A
Grayscale

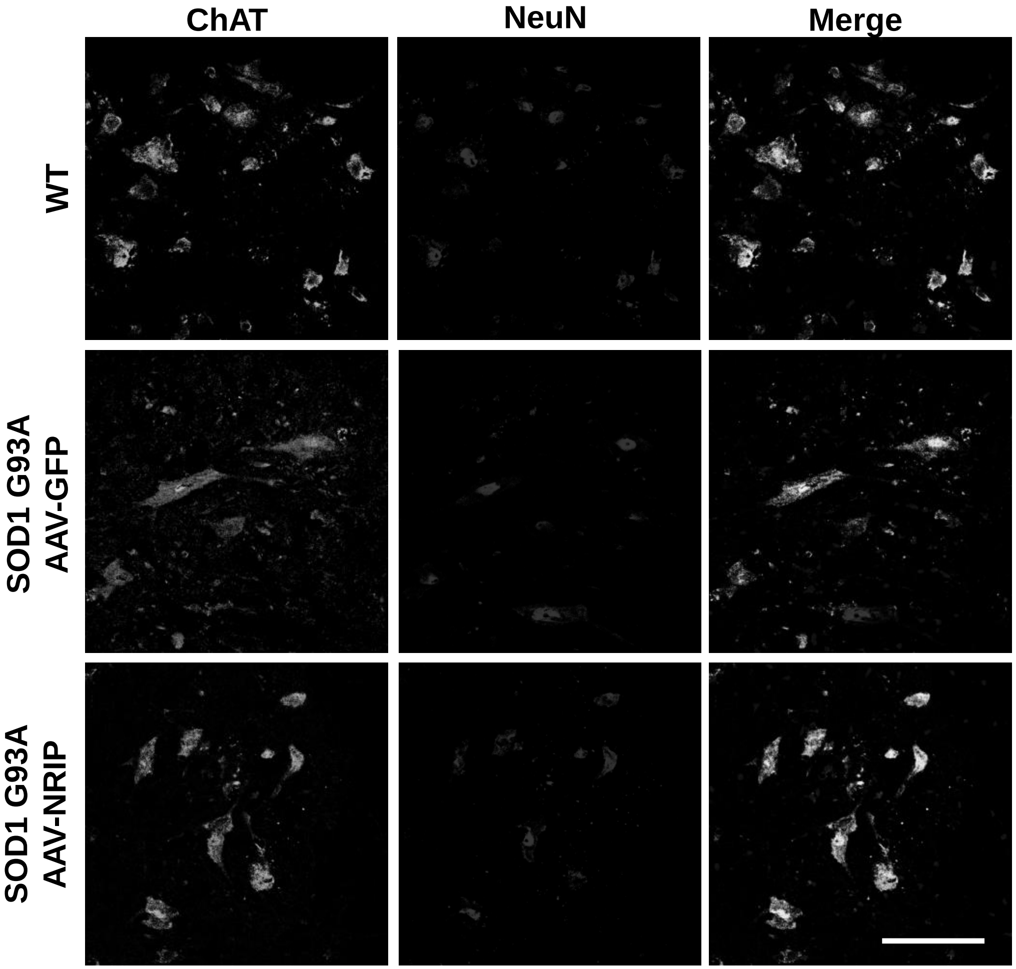

Figure S2
Grayscale

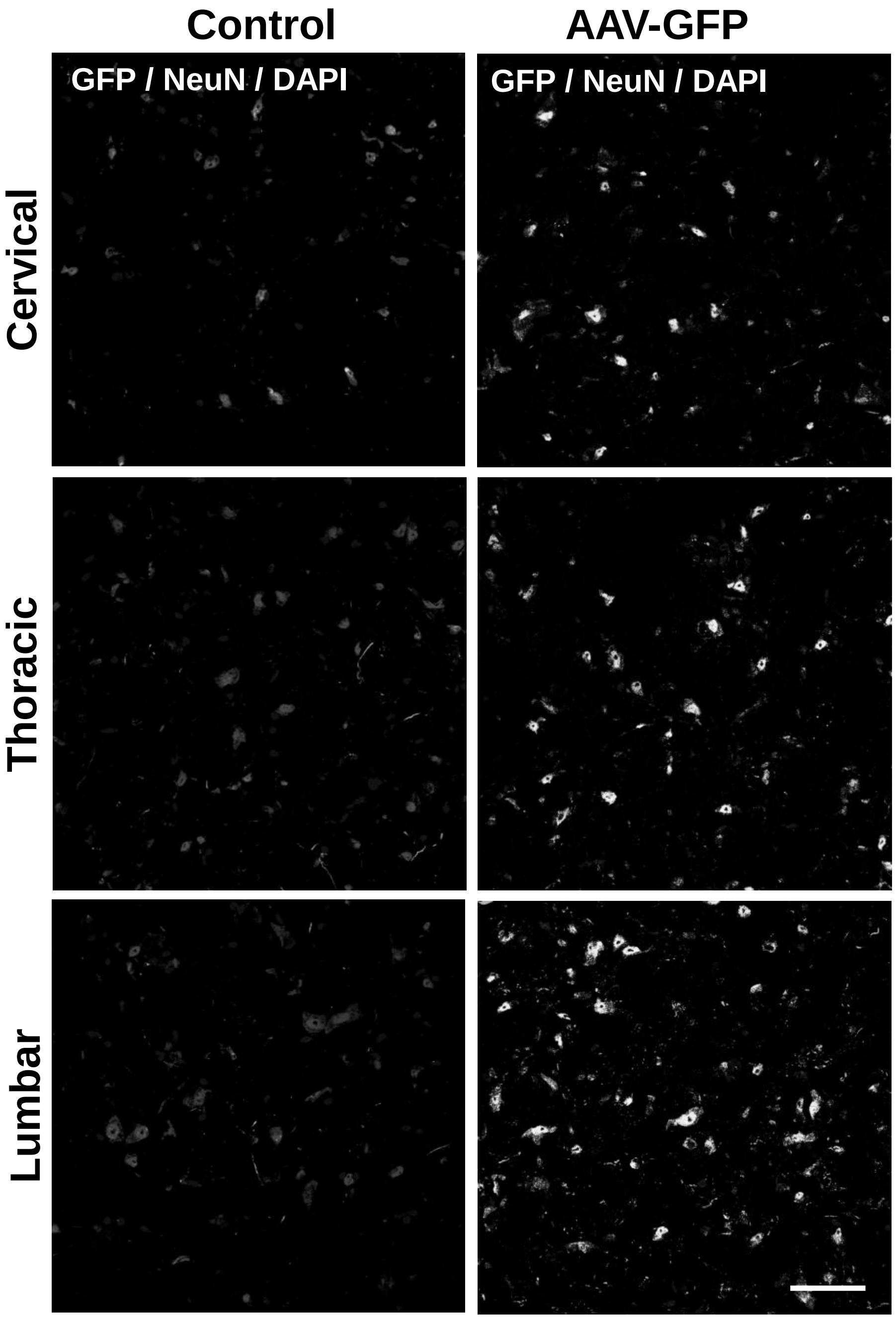

Supplement: Supplementary file 1 — Supplementary Material 1. [file 13395_2024_349_MOESM1_ESM.pdf]
